# Treatment of flexibility of protein backbone in simulations of protein–ligand interactions using steered molecular dynamics

**DOI:** 10.1038/s41598-024-59899-3

**Published:** 2024-05-07

**Authors:** Duc Toan Truong, Kiet Ho, Dinh Quoc Huy Pham, Mateusz Chwastyk, Thai Nguyen-Minh, Minh Tho Nguyen

**Affiliations:** 1 Laboratory for Chemical Computation and Modeling, Institute for Computational Science and Artificial Intelligence, Van Lang University, Ho Chi Minh City, 70000 Vietnam; 2 Faculty of Applied Technology, School of Technology, Van Lang University, Ho Chi Minh City, 70000 Vietnam; 3 Institute for Computational Science and Technology (ICST), Quang Trung Software City, Ho Chi Minh City, 70000 Vietnam; 4 Institute of Physics, Polish Academy of Sciences, Warsaw, Poland; 5 University of Medicine and Pharmacy at Ho Chi Minh City, Ho Chi Minh City, 70000 Vietnam

**Keywords:** Protein–ligand complexes, Protein flexibility, Ligand affinities, Ligand release, Steered molecular dynamics simulations, Restrain modes, Biochemistry, Biophysics, Computational biology and bioinformatics, Chemistry

## Abstract

To ensure that an external force can break the interaction between a protein and a ligand, the steered molecular dynamics simulation requires a harmonic restrained potential applied to the protein backbone. A usual practice is that all or a certain number of protein’s heavy atoms or Cα atoms are fixed, being restrained by a small force. This present study reveals that while fixing both either all heavy atoms and or all Cα atoms is not a good approach, while fixing a too small number of few atoms sometimes cannot prevent the protein from rotating under the influence of the bulk water layer, and the pulled molecule may smack into the wall of the active site. We found that restraining the Cα atoms under certain conditions is more relevant. Thus, we would propose an alternative solution in which only the Cα atoms of the protein at a distance larger than 1.2 nm from the ligand are restrained. A more flexible, but not too flexible, protein will be expected to lead to a more natural release of the ligand.

## Introduction

Since its first introduction in 1986^1,2^, the atomistic force microscopy (AFM) has provided us with a wealth of information about the mechanical properties of ligand–protein structures. Despite some fundamental limitations that have not yet been overcome^3,4^, the AFM technique remains a valuable tool for examining how a macromolecular target is assembled^5–8^.

In a typical AFM experiment, a spring is employed to attach a macromolecular system. In this setup, the spring is moved away from the anchored molecule at a chosen speed, generating an external force that not only affects the biological system but also allows to extract its kinetic information. Study on how proteins and ligands unbind from each other at an atomistic level is particularly interesting because these observations bring in crucial information to the field of rational drug design, including, among others, the unbinding pathways, residence time, and dissociation rate^9,10^.

The unbinding process of a ligand–protein complex continuously occurs over a large timescale, ranging from microseconds to several seconds^11^. Although current methodologies have expanded our knowledge, experimental study of this process remains a challenging task. Therefore, for a comprehensive understanding, scientific research is increasingly drawn to computational approaches that have recently played a significant role in this area thanks to rapid advances in both technical hardware and software^12,13^.

To replicate the principles of atomic force microscopy (AFM) experiments in silico, the steered molecular dynamics (SMD) simulation which was first developed in 1996^14–18^, has often been employed. This computational approach allows the ligand to play the role of the linker molecule, and the protein acts as the anchored one^19^.

A conventional SMD protocol carefully selects a pulling direction based on structural information^20–22^. To complete a dependent trajectory the external force must successfully drive the ligand far away from the protein's active site. During this motion, both the force–time profile and displacement–time profile are recorded. Numerous SMD applications have been reported in which it cannot only be applied to generate a ligand–protein unbinding process, but also to evaluate the binding affinity following the principle that 'the larger the rupture force, the higher the binding affinity will be'^21,23–27^. Steered molecular dynamics simulation also plays a crucial role in bioinformatics analysis and rational drug design^28–30^.

Notably, to ensure that the ligand is pulled out of the protein interactive space, a harmonic potential needs to be applied to the protein backbone to restrain its motion. This adaptation prevents the external force from applying too much on the receptor; otherwise, the SMD performance may induce a steady drift in the water solution rather than focusing on the breaking of the ligand–protein interaction.

Grubmuller and Schuteln were known as pioneers in implementing SMD simulations^14^. In a study published in 1996, this group fixed all of the protein's atoms including the C, N, S and O atoms^14^. In two subsequent studies in 1997 and 1999, Schuteln’s research group constrained all the Ca atoms on top of the human retinoic acid receptor^16^ (hRAR protein) and restrained the mobility of six C atoms of the bacteriorhodopsin protein^31^.

Other methods used by Schuteln's group included the holding the center of mass (COM) of all the carbon atoms in the neuraminidase protein^32^, fixing carbon atoms of the residues 26, 31, 56, 215, 307 and 333 at the front face of the Gelsolin segment-1 protein^33^. Since SMD has proved to be a promising tool^34^ due to its fast computing, friendly implementation but with high accuracy, numerous restrained methods have been reported^24,35^. However, the way that previous authors restrained the protein's mobility have been found to be not consistent in going from one study to another^24,32–35^.

In more recent studies, some authors still preferred restraining the motion of all heavy atoms, while others restrained a small group of atoms. A brief summary given in Table 1 lists a part of these inconsistencies. It is a challenging task for us to adequately summarize all related studies reported in the last three decades; it is rather the work of a comprehensive review which goes beyond the scope of the present study. According to our best summarization, when performing a SMD simulation, one of three constrained techniques has usually been employed, namely (1) fixing all heavy atoms of the protein; (2) fixing all alpha-carbon atoms of the protein, and (3) fixing some chosen Cα atoms depending on specific purposes. Moreover, in some protocols, the authors omitted to share the necessary information of their chosen method for reproduction^35,36^. It appears that a harmonic potential has been applied to the simulation without any rational or justification.

**Table 1:** A summary of different restrain methods employed in the steered molecular dynamics simulation during the last three decades.

Nr	Year	Complex	Fixed atoms	PDB ID
1	1997	Bacteriorhodopsin and retinal (C_20_H_28_O)	Six atoms, the Cα—atoms of six residues, at the ends of helices A, B, and C^16^	1BRD
2	1999	Human retinoic acid receptor hRAR and retinoic acid (C_20_H_28_O_2_)	Set of atoms, all of Cα—atoms in the top part of the protein^31^	2LBD
3	1999	Gelsolin sesgment 1 and Adenosine-5'-triphosphate (C_10_H_16_N_5_O_13_P_3_)	Set of atoms, all carbon atoms of residues 31, 26, 56, 215, 307, and 333 at the front face of the protein^33^	1EQY
4	2002	Acetylcholine receptors and huperzine A (C_15_H_18_N_2_O)	Center of mass of AChE protein^37^	1VOT
5	2003	HIV-1 reverse transcriptase and (2-acetyl-5-methylanilino)(2,6-dibromophenyl)acetamide (C_17_H_16_Br_2_N_2_O_2_)	All heavy atom^38^	1HNI
6	2005	Acetylcholine receptors and 1-benzyl-4-[(5,6-dimethoxy-1-indanon-2-yl)methyl]piperidine (C_24_H_29_NO_3_)	None report^39^	1EVE
7	2006	Acetylcholine receptors and acetylcholine (C_7_H_16_NO_2_^+^)	One atom, a Cα of residue V-109^40^	1I9B
8	2010	Neuraminidase and oseltamivir carboxylate (C_14_H_24_N_2_O_4_)	Center of mass of protein Cα—atoms^32^	2HU4, 3CL0, 3CL2
9	2015	Thrombin and C_32_H_35_BrN_2_O_2_S, neuraminidase and C_11_H_19_NO_9_, penicillopepsin and alpha-D-mannopyranose (C_6_H_12_O_6_)	Set of atoms, Cα—atoms of the receptor which are 3 Å behind the last atom of the ligand^41^	1D3D, 1NSC, 1APT
10	2015	Bovine beta-lactoglobulin and octanoic acid (C_8_H_16_O_2_)	Cα—atoms far from binding site are fixed^42^	3NQ9
11	2019	Human Adenosine A2A Receptor and C_23_H_29_N_7_O_6_, C_16_H_15_N_7_O_2_	Five atoms, Cα—atoms of five residues at the top of the transmembrane helices (residues 9, 80, 177, 256 and 270)^43^	4UHR, 5IU4
12	2020	Trypsin and benzamidine (C_7_H_8_N_2_)	All Cα—atoms^44^	3PTB
13	2020	Human Abl kinase domain and imatinib (C_29_H_31_N_7_O)	All backbone atoms of protein’s binding pocket^45^	2HYY
14	2020	RDB of Spike protein and simeprevir (C_38_H_47_N_5_O_7_S_2_), or lumacaftor (C_24_H_18_F_2_N_2_O_5_)	All atoms of protein backbone^46^	6LZG
15	2021	Heat shock protein 90 (Hsp90) and C_13_H_10_BrN_5_	All Cα—atoms^47^	3K99
16	2022	Spike protein of SAR-2 virus and ACE2 and silodosin (C_25_H_32_F_3_N_3_O_4_)	Cα—atoms of residues 519, 333, 360, 525, 386 and 517.^48^	6LZG
17	2022	Neuraminidase and capsaicin (C_18_H_27_NO_3_)	All Cα—atoms^49^	2HU0

The flexibility of macromolecules has become a fundamental component that must be considered in computational modeling, especially in computational aided drug design^50–54^. Additionally, as more crystal structures of protein–ligand complexes have been recorded, it is found that a ligand can bind to different conformations of the same receptor. These differences are induced by specific structural rearrangements of one or more amino acids located in a narrow region of the protein's active site. The flexibility of these residues predominantly affect the ligand’s behavior when that ligand is released from a crowded region^55–57^. However, this important aspect in steered molecular dynamic simulation has not received much attention.

In a SMD study, Zhang^58^ changed the k-constant of the restrained harmonic potential to explore the influence of protein flexibility. This author demonstrated that a lower harmonic force applied to all C-atoms of the protein results in a larger variety of protein–ligand conformations. Except for Zhang’s study, to our best knowledge, no additional information on this aspect is available. Such an absence has indeed led to a deeper inconsistency when various restrained methods are still used in SMD simulations, as mentioned in the paragraph above (cf. Table1). Evidence from Zhang's study has clearly warned that a rigid fixing of all heavy atoms or all Cα atoms could neglect the contribution of protein motion to the unbinding process. In contrast, from the opposite viewpoint, we are concerned that a too weak restrained force or a too flexible protein is not be able to stop the drifting of the whole structure^59,60^. A legitimate question is what could happen when the external force focuses on stretching the protein rather than on rupturing the ligand–protein interaction.

Another issue is as to whether the entire ligand–protein complex drifts under the influence of the water bulk layer. In this context, the questions of interest that motivate the present study is how the protein motion could be restrained in SMD simulations, and what differences could be expected by these different methods.

To investigate the differences in relevant approaches, we are developing various restricted ways before applying them to the same ligand–receptor complex. To take these ways into account, we propose six parallel SMD simulation systems in which six different groups of Cα atoms of a protein are held. Unlike the previous study of Zhang^58^ where the author reduced the k-constant of the harmonic potential from 1000 to 5 kcal/mol.nm^2^, we relax in the present study the protein by narrowing the portion of the protein backbone where a restrained harmonic potential could be applied. Each of the six independent preparations is described in detail in the following section.

Under the influence of six different approaches, changes in the protein's geometric structure are expected to be observed. The ligand–protein complex chosen from the PDB Bank needs to satisfy the following conditions, namely, (a) the protein has a wide enough tunnel to prevent the ligand from collapsing the protein gorge; (b) no metallic atoms are present in the protein binding pocket^61–63^ and (c) only one protein chain has the ability to carry out the protein’s function. During the output data collection, the intermolecular interaction between protein and ligand is calculated, including the number of contacts and the number of hydrogen bonds. The activity of every major residue located near the active site is carefully explored. The force–time dependence and the displacement–time dependence are monitored, in a similar way as in previous traditional SMD simulations. A non-equilibrium process with a smaller unbinding barrier would represent a state closer to an equilibrium process. The most sufficient way of using a restrained harmonic potential could be introduced for further investigation aiming to demonstrate the ligand–protein unbinding pathway by SMD simulation.

## Materials and methods

### System preparations

The PDB Bank provides atomistic structures of ligand–protein binding complexes. Ligand-contained proteins are sourced from the PDB Bank, the relevant literature, and previous reviews, etc. Six proteins including the ones noted as PDB-IDs 4JNJ, 2JFZ, 1PYE, 1TSL, 2YDV, 1EVE are selected. Before adding hydrogen atoms using the Gromacs software (version 2020^64^), missing residues are repaired with Pymol Software^65,66^. The Amber ff99SB-ILDN force field is implemented. The Gaussian 16^67^ package is used to optimize geometry structure of the ligands and subsequently to determine the charge distribution. The ligand’s conformations are optimized and its electrostatic potential maps are calculated at the B3LYP/6–31 + G(d,p) level. Atomic net charges of the ligands are derived using the RESP^68^ method. The Antechamber module of AMBER Tools is applied to calculate additional parameters for the compounds using the General Amber Force Field (GAFF)^69,70^. Simulated cubic boxes are set to ensure a distance greater than 0.6 nm between the protein surface and boundaries. After solvating in water, Na or Cl ions are added to neutralize the system. Particle mesh Ewald (PME)^71^ method is used for long-range electrostatic interactions, and periodic boundary conditions are set. The SHAKE^72^ algorithm is applied to covalent bonds of hydrogen atoms. The non-bonded contact between two atoms is avoided when the pair distance is larger than 1.0 nm. The system preparation is similar to our previous setup.^24^ The tables mentioned above, (Table 2 and Tables S1 and S2 in the Supplementary Information file (SI)) list the PDB-IDs of the complexes utilized. Pulling directions are identified based on the support of the Carver web server version 1.0 and aligned to the Z—axis of coordinate systems^73^.

**Table 2:** General information of simulation.

PDB-ID	4JNJ	2JFZ	1PYE	1TSL	2YDV	1EVE
Name	Streptavidin monomer	Glutamate racemase	Cyclin dependent kinase	Bacterial thymidylate synthase	Adenosine A2 receptors	Acetylcholinesterase AChE
(a) General information of protein—ligand system						
Protein's number residues	115	255	298	316	325	534
System's number atoms	26.860	37.453	43.552	52.649	64.288	89.132
(b) Number of Carbon alpha atoms be fixed in a restrained method						
PDB-ID	4JNJ	2JFZ	1PYE	1TSL	2YDV	1EVE
Mode 1	774	1758	1885	2278	1923	3730
Mode 2	115	255	298	316	325	534
Mode 3	49	173	134	201	120	304
Mode 4	44	161	85	183	119	234
Mode 5	21	58	22	68	44	69
Mode 6	5	27	3	41	22	44

### Restraining method

**Figure 1:**
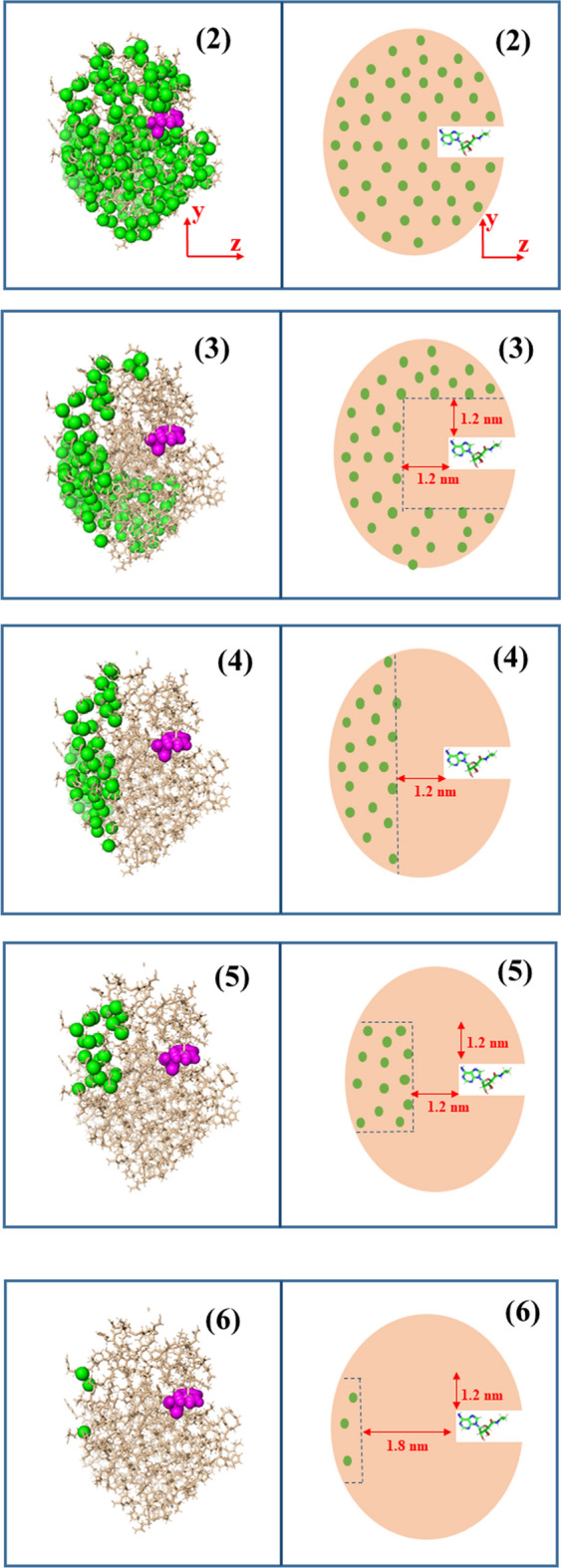
Cartoon mapping of five restrained modes from 2 to 6. Representative group of atoms of CDK-2 protein, PDB-ID 1PYE (lower). Y–Z directions are shown in (2) (restraining all heavy atoms not shown). Small images are arranged from 2 to 6, respectively, with increasing protein flexibility.

The present study aims to determine the influence of different restrained methods on the results obtained by conventional steered molecular dynamic simulations. Six ways of fixing protein backbone are prepared: (1) fixing all heavy atoms of protein (C, N, S, O); (2) fixing all Cα atoms of protein; (3) fixing all Cα atoms with a distance to ligand greater than 1.2 nm; (4) fixing all Cα atoms with the perpendicular distance in the pulling direction to ligand greater than 1.2 nm; (5) fixing all Cα atoms with the perpendicular distance in the pulling direction to ligand greater than 1.2 nm and all Cα atoms with the perpendicular distance in the x–y direction to ligand smaller than 1.2 nm, and (6) fixing all Cα atoms with the perpendicular distance in the pulling direction to ligand greater than 1.8 nm and all Cα atoms with the perpendicular distance in the x–y direction to ligand smaller than 1.2 nm. Six groups of protein atoms are presented in Fig. 1. In the discussion hereafter, each way of fixing is named as mode, from mode 1 to mode 6 corresponding to the six fixing conditions defined above.

### Steered molecular dynamics simulations

Some of the complexes selected here have already been examined in previous studies^39,74^. However, creating uniformity within existing results is not possible due to large variations in parameters including force field, pulling velocity, and the pulling direction-defined method… implemented in those protocols. Therefore, simulations with a consistent set of parameters are carried out. In our protocol, a hundred independent trajectories will be performed in each mode of the restrained method. A constant pulling velocity (v = 1.0 nm/ns) and a spring constant (k = 600 kJ/mol/nm^2^) are chosen. Snapshots are saved every 5000 steps (2 fs for each step). The duration time of pulling is set to 3 ns to ensure that all ligands are pulled far away from the protein. In each trajectory, the time-dependent force/displacement is recorded every 10 fs. External pulling work and unbinding barrier free energy are computed using the protocol defined in our previous studies^24^.

### Hydrogen bond and contact

When a protein's heavy atom has the smallest distance to one atom of the ligand, being less than 0.6 nm, an intermolecular contact is formed. If the acceptor–donor distance is less than 0.35 nm and the acceptor-hydrogen-donor angle is greater than 135^0^, a hydrogen bond is considered available. Tasks are performed using the Gromacs packages including gmx hbond and gmx mindist. All hydrogen bonds created between inhibitors and the receptors are taken into account. In each snapshot of an independent SMD trajectory, the number of hydrogen bonds and the ligand's center of mass (COM) are analyzed. Following this way, we can average the number of hydrogen bonds depending on the position of the COM.

### Protein root mean square deviation

The root mean square deviation (RMSD) of the protein is calculated as the dissimilarity of all atom coordinates to its initial structure in three dimensions. These analytical procedures are carried out using the gmx_rms tool supported in the Gromacs package, and the appropriate formula is as follows:

$$RMSD(x,x^{ref} ) = \sqrt {\frac{1}{N}\sum\limits_{i = 1}^{N} {\left| {(x_{i} - x_{i}^{ref} } \right|^{2} } }$$

where N is the number of atoms of the protein, and x represents the three-dimensional coordinate.

## Results and discussion

### Convergence of numerical data and distorted Gaussian-type distribution of values

**Figure 2:**
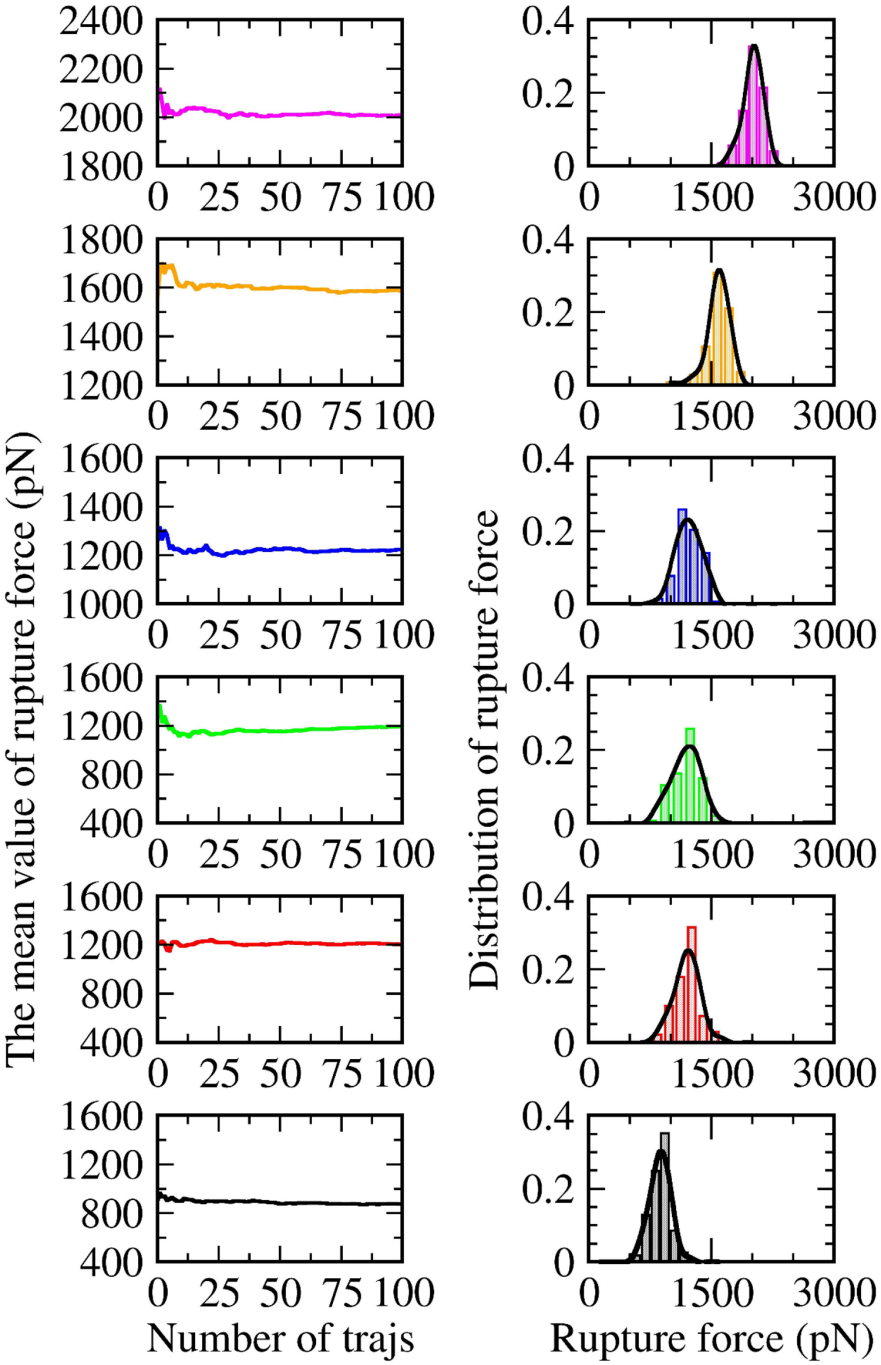

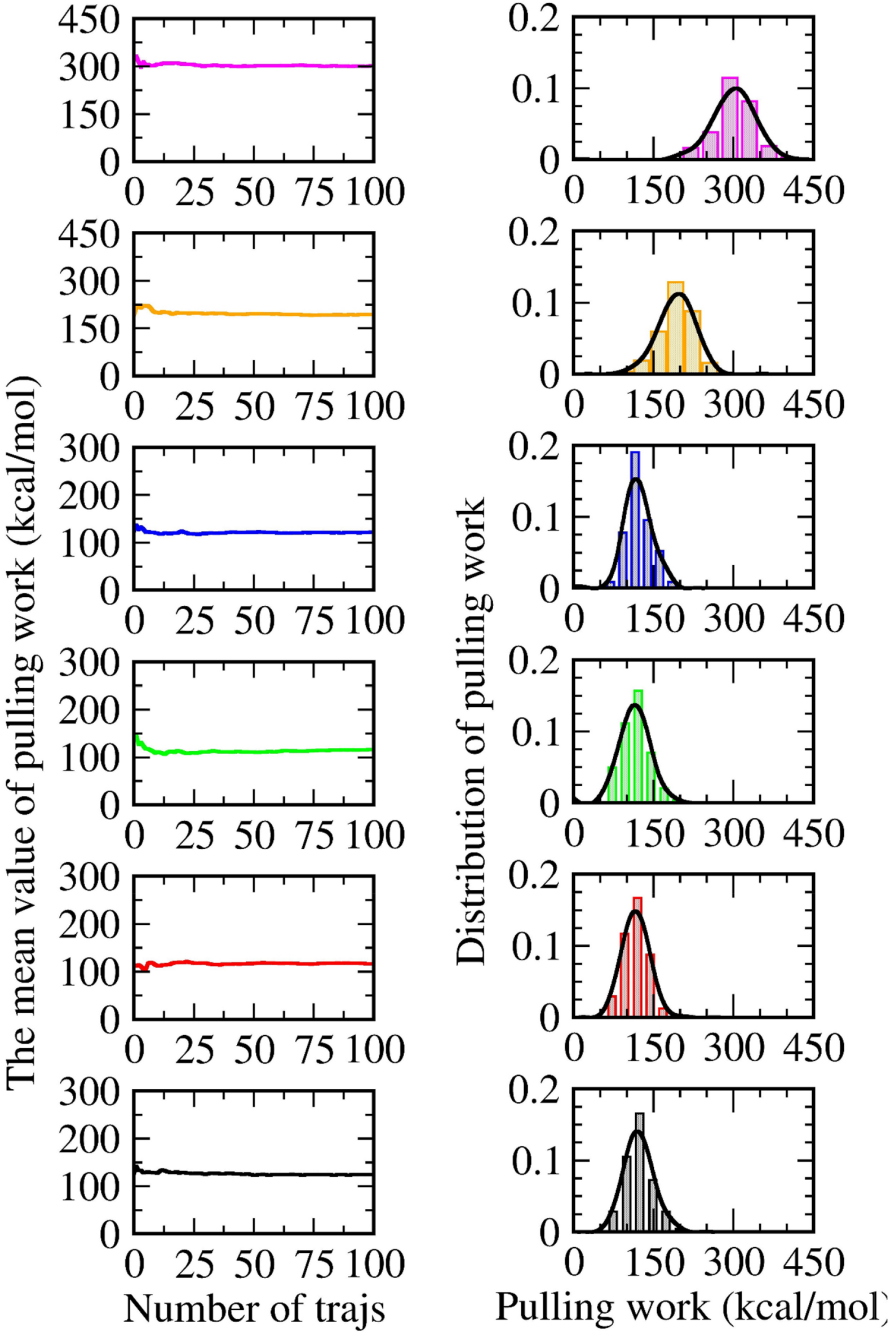
The mean value of rupture force (left) and external work (right) dependent on the number of pulling trajectories. Data are obtained from six restrained modes of the 4JNJ system, with 100 independent pulling trajectories in each mode: mode 1 (in magenta), mode 2 (in orange), mode 3 (in blue), mode 4 (in green), mode 5 (in red), and mode 6 (in black). The distribution of rupture force (middle—right) and the distribution of pulling work (right) show a distorted Gaussian curve (black line).

Every physical quantity considered in this study is derived from a non-equilibrium process. Due to our limited computing power, we could only apply a fast growth evolution when the pulling velocity is chosen at 1 nm/ns. In replicating the results of the AFM experiment and analyzing the SMD data, we monitor the time-dependent force and record the maximum value, known as ${{\text{F}}}_{\text{max}}$, right after a rupture event occurred. To achieve the outcome convergence, previous studies have indicated that steered molecular dynamics (SMD) simulations require a suitable number of independent trajectories, neither too large nor too small. In the context of using a relatively small velocity (v = 1 m/s), although there are minor differences in the number of trajectories each system required to reach result convergence, we recognize that about 100 orbits are sufficiently large for all of them. Figure 2 serves as an example, including the rupture force and external work in dependence on the number of pulling trajectories. Accordingly (Fig. 2), data convergence is found in every restraint mode, from mode 1 to mode 6, and in every ligand–protein complex (data not shown).

### Force–time and work-time profile obtained from different restrained modes of the ligand release

All values of $\langle {{\text{F}}}_{\text{max}}\rangle$ and $\langle {{\text{W}}}_{\text{pull}}\rangle$ are listed in Table 3. Overall, mode 1 and mode 2 of every system are consistently held the first and second positions, significantly larger than the others. The mean values of rupture force and pulling work from 100 trajectories of each pulling mode are taken into account, as shown in Table 3. Restraining all heavy atoms revealed values of 983 pN, 1440 pN, 1594 pN, 2010 pN, 1147 pN, 917 pN for $\langle {{\text{F}}}_{\text{max}}\rangle$ and 90.5, 160.1, 210.2, 301.0, 140.9 and 87.4 kcal/mol for $\langle {{\text{W}}}_{\text{pull}}\rangle$, in the cases of 1EVE, 2JFZ, 2YDV, 4JNJ, 1PYE and 1TSL systems, respectively. In the case of restraining all carbon atoms, $\langle {{\text{F}}}_{\text{max}}\rangle$ is decreased to values of 694 pN, 688 pN, 833.7 pN, 1590 pN, 721 pN, 523 pN, and $\langle {{\text{W}}}_{\text{pull}}\rangle$ lowered to 58.1, 47.2, 96.2, 193, 63.1, and 38.2 kcal/mol, respectively. In summary, based on the analysis of $\langle {{\text{F}}}_{\text{max}}\rangle$ and $\langle {{\text{W}}}_{\text{pull}}\rangle$, we recognize that our six fixing methods may be classified into two main classes: (A) rigidly fixing including mode 1, mode 2 and B) flexibly fixing including mode 3 to mode 6. When compared to the corresponding quantities in four flexible modes, the mean values of rupture forces and pulling works in two rigidly fixing methods of class A are outstandingly higher.

**Table 3:** The averaged value of rupture force (a), pulling work (b), unbinding barrier (c) and root-mean-square deviation (d) obtained from 100 independent trajectories.

PDB-ID	4JNJ	2JFZ	1PYE	1TSL	2YDV	1EVE
(a) Rupture force (pN)						
Mode 1	2010 ± 12	1440 ± 5	1147 ± 24	917 ± 6	1594.8 ± 8.7	983 ± 4
Mode 2	1590 ± 14	688 ± 8	721 ± 13	523 ± 7	833.7 ± 8.6	694 ± 8
Mode 3	1214 ± 14	473 ± 7	537 ± 11	377 ± 6	539.7 ± 11.3	629 ± 7
Mode 4	1192 ± 15	453 ± 8	499 ± 9	344 ± 7	530.7 ± 10	608 ± 7
Mode 5	1200 ± 14	422 ± 7	447 ± 11	344 ± 6	537.6 ± 7.7	597 ± 7
Mode 6	874 ± 11	398 ± 8	411 ± 10	337 ± 6	523.1 ± 9	605 ± 7
(b) Pulling work (kcal/mol)						
Mode 1	301 ± 3	160.1 ± 1.1	140.9 ± 3.3	87.4 ± 0.9	210.3 ± 1.8	90.5 ± 0.6
Mode 2	193 ± 3	47.2 ± 0.7	63.1 ± 1.4	38.2 ± 0.8	96.2 ± 1.4	58.1 ± 0.8
Mode 3	122 ± 2	30.4 ± 0.6	48.7 ± 1.6	25.2 ± 0.8	53.5 ± 1.3	52.8 ± 0.8
Mode 4	115 ± 2	27.8 ± 0.6	44.1 ± 1.5	18.5 ± 0.9	52.6 ± 1.3	51.2 ± 0.8
Mode 5	116 ± 2	26.7 ± 0.6	32.3 ± 1.5	21.2 ± 0.9	55.2 ± 1.1	51.9 ± 0.9
Mode 6	124 ± 2	25 ± 0.7	30 ± 1.5	21.6 ± 0.9	54.6 ± 1.4	54.8 ± 0.9
(c) Unbinding barrier (kcal/mol)						
Mode 1	290.3 ± 3.5	147.2 ± 1.1	99.3 ± 4.2	45.4 ± 0.7	168.1 ± 1.9	60.2 ± 0.6
Mode 2	179.6 ± 3.1	35.4 ± 0.8	37.2 ± 1.5	15.7 ± 0.5	49.8 ± 1.1	27.3 ± 0.7
Mode 3	106.7 ± 2.4	16.6 ± 0.5	19.4 ± 0.8	10.3 ± 0.4	21.5 ± 1.2	21.8 ± 0.6
Mode 4	101.9 ± 2.6	15.9 ± 0.6	17.1 ± 0.8	9.2 ± 0.4	20.7 ± 0.9	21.7 ± 0.6
Mode 5	102.7 ± 2.3	13.8 ± 0.5	15.0 ± 0.7	8.4 ± 0.3	20.5 ± 0.7	18.9 ± 0.7
Mode 6	24.6 ± 0.6	12.5 ± 0.5	12.1 ± 0.5	8.2 ± 0.3	19.5 ± 0.7	18.1 ± 0.6
(d) Averaging RMSD value (A^0^) of protein backbone at final SMD step						
Mode 1	0.247 ± 0.001	0.231 ± 0.001	0.211 ± 0.001	0.226 ± 0.001	0.229 ± 0.001	0.226 ± 0.001
Mode 2	0.405 ± 0.003	0.407 ± 0.002	0.428 ± 0.002	0.411 ± 0.002	0.422 ± 0.002	0.396 ± 0.001
Mode 3	0.84 ± 0.02	0.73 ± 0.01	0.89 ± 0.02	0.92 ± 0.023	1.22 ± 0.02	0.663 ± 0.006
Mode 4	0.99 ± 0.02	0.83 ± 0.01	0.975 ± 0.026	1.68 ± 0.04	1.22 ± 0.02	1.01 ± 0.01
Mode 5	1.0 ± 0.02	1.15 ± 0.02	1.17 ± 0.03	2.04 ± 0.06	1.64 ± 0.02	1.19 ± 0.01
Mode 6	1.85 ± 0.07	1.32 ± 0.02	1.51 ± 0.03	1.91 ± 0.06	1.76 ± 0.03	1.22 ± 0.01

**Figure 3:**
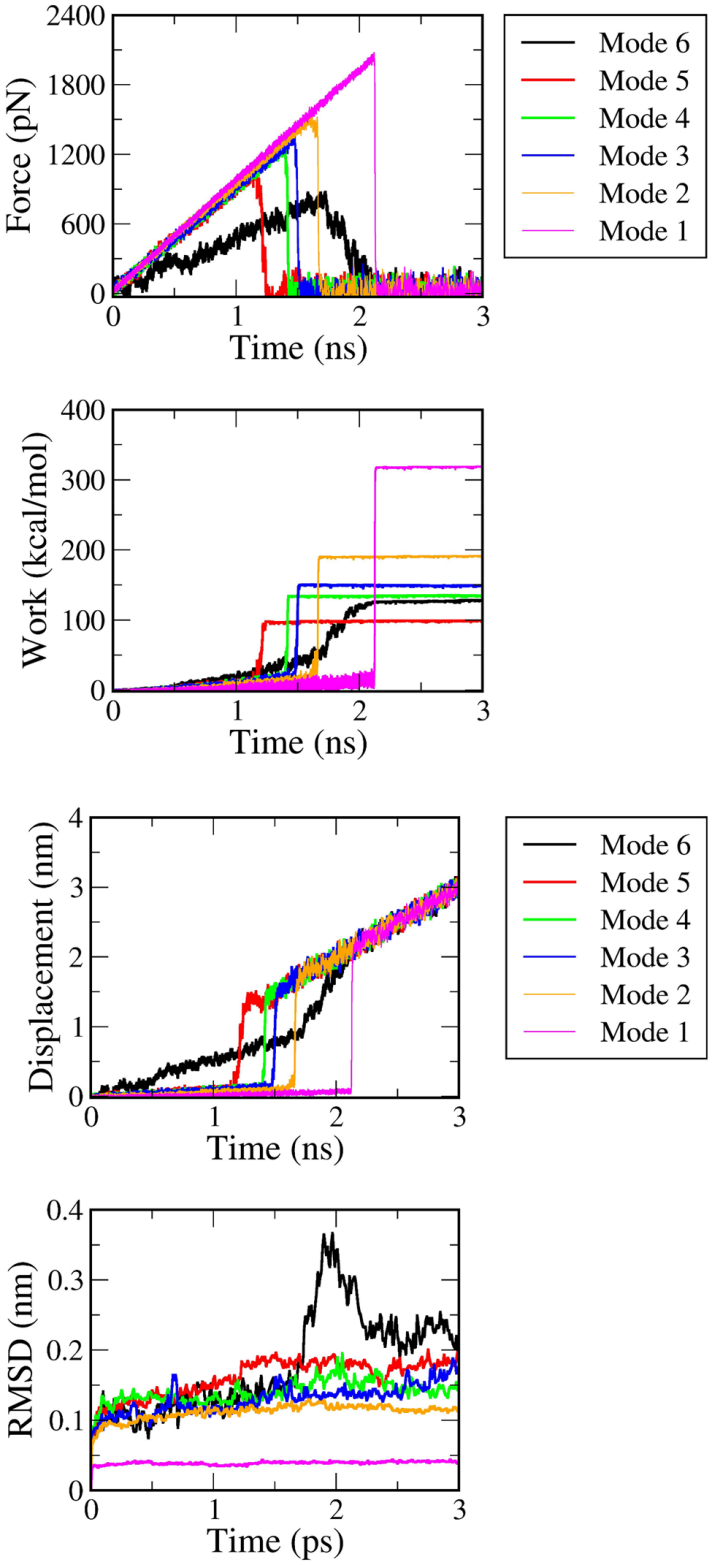
Time-dependent force (upper-left) and time-dependent ligand's displacement (upper-right), time-dependent work (lower-left), and time-dependent RMSD (root mean square deviation) (lower-right). Results are randomly plotted from six representative trajectories under six different restrained methods of the 4JNJ system. Averaged values of rupture force and pulling work, obtained over 100 trajectories in six systems, are shown in the SI file.

Over the past two decades, there has been much discussion about how a pulling rate^14,75,76^ influences the rupture force and external work of a non-equilibrium process. There is a consensus that a decrease of the pulling rate does not only decrease the rupture force and the external work but also push the non-equilibrium process to evolve into an equilibrium one. In other words, the smaller the value we obtain, the faster the system reaches an equilibrium. According to this knowledge, neither mode 1 nor mode 2 is expected to attain a sufficient way to generate a natural process of ligand–protein dissociation. This information could normally be predicted, but our evidence serves as a useful warning for an eventual selection of mode 1 or mode 2 in a SMD simulation. Figure 3 shows the time-dependent pulling force, displacement, RMSD, and external work in a representative trajectory from pulling the 4JNJ system. The averaged lines from 100 trajectories are shown in Figs. S1–S6 of the SI file.

In the paragraph above, we mention that the modes in class A have stronger rupture forces and larger pulling works, that are outstanding as compared to the outcomes attained by the approaches in class B. Here we are going into more detail about the results of simulations generated using lighter restraining approaches, from mode 3 to mode 6. Collected data in almost all cases, listed in Table 3, seem to interpret two common understandings, namely, i) the more flexible the protein is, the smaller the rupture force will be, and ii) the more flexible the protein is, the lower the pulling work the external force will perform.

When examining the 1EVE system, the mean rupture forces in modes 4, 5 and 6 are measured at values: 608 ± 7 pN, 597 ± 7 pN, 605 ± 7 pN. Similarities are also detected in the 2YDV system and 4JNJ system, modes 3, 4 and 5; in 1TSL system, modes 4, 5and 6. A question of concern is as to whether there is any misinformation. It is reasonable to see that these equivalent values of F_max_ are caused due to the geometric feature of protein structure where some distinct modes have restrained some quite similar groups of Cα atoms. Protein 1EVE and 1TSL are formed from globulin structures while 2YDV and 4JNJ are formed via beta-barrel structures. Data collected from our simulations truly confirm the first statements given above. To prove the second statement, we now calculate the pulling work. 

Looking now at mode 6 in all systems considered, although six systems create the smallest value of averaging rupture forces $\langle {{\text{F}}}_{\text{max}}\rangle$, the mean value of pulling work $\langle {{\text{W}}}_{\text{pull}}\rangle$ is not always the lowest one. There are 4/6 protein–ligand complexes like that. The systems 1EVE and 4JNJ, under mode 6 conditions, have received the mean values $\langle {{\text{W}}}_{\text{pull}}\rangle$ of 54.8 kcal/mol and 124 kcal/mol, respectively. These results are higher than the mean value of pulling work in mode 5, which are 51.9 kcal/mol and 116 kcal/mol.

To investigate these issues, we plot in Fig. 3 four curves including the time-dependent force, the time-dependent displacement, the time-dependent work, and the time-dependent root-mean-square deviation (RMSD) from one representative trajectory in the 4JNJ system. According to the RMSD black line (in Fig. 3), it is rather easy to recognize that the ligand leaves its initial position immediately when the external force starts increasing. The averaged value of RMSD of the protein backbone is collected in Table 3. Since mode 5 and mode 6 usually own a higher amount of averaged RMSD, this raises a suspect: instead of breaking the protein–ligand dissociation, the external force aims to stretch the protein and induce an unnecessary value of pulling work. More seriously, we also find in some cases of mode 6: the bulk water layer induces the protein to spin perpendicularly to the pulling direction. The ligand thereby collapses to the protein wall as captured in Fig. S25 (SI file). Although waste trajectories are not appeared frequently and are manually ejected in this examination, this leads to more concerns when using mode 6 or even mode 5 in the steered molecular dynamic simulations.

### Flexibly restraining mode lowers the unbinding barrier for ligand crossing

**Figure 4:**
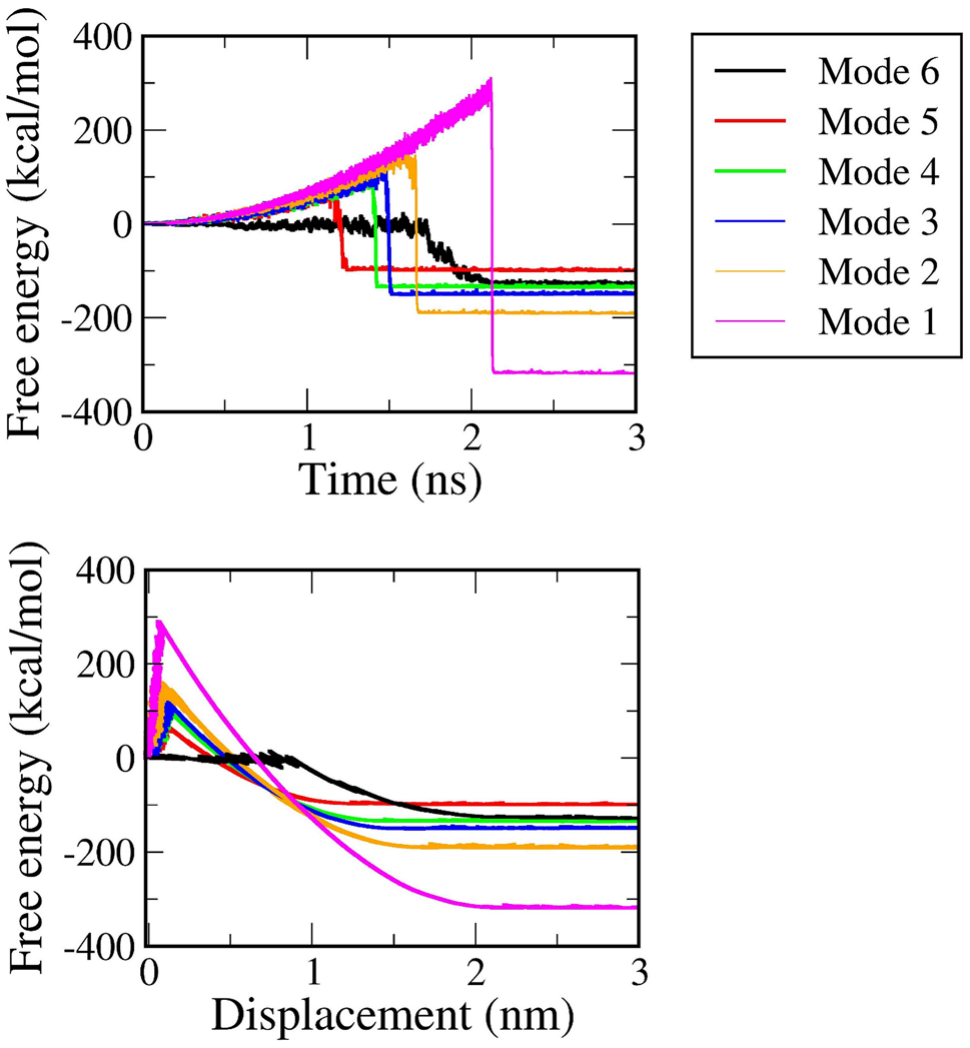
Free energy profile of six representative trajectories when a ligand was pulled out of the streptavidin protein, PDB ID 4JNJ. Results are obtained under six restrained methods.

In the biophysical literature, an unbinding process is usually conceptualized as a barrier crossing in which the system is transformed from a higher free energy conformation to a lower. Thus, we now construct the free energy profile of a relevant non-equilibrium kinetic process^22^. The unbinding free energy barriers of six 4JNJ representative trajectories are plotted in Fig. 4. Collected results show that if all heavy atoms of the protein are constrained, ligands must overcome the highest energy barrier to successfully escape far away from the protein.

Every complex clearly displays the remarkable values of mode 1 and mode 2 unbinding barriers which are listed in part (c) of Table 3. In the case of 1EVE, a globulin protein complex, two highly fixing strategies, all heavy atoms mode 1 and all Cα atoms mode 2, give the mean values of 60.2 kcal/mol and 27.3 kcal/mol, respectively, whereas the mode 6 restrained method only produces an unbinding barrier of 18.1 kcal/mol. All other protein–ligand complexes likewise exhibit a notable variation between the unbinding barrier's lowest value and its two greatest values. The ligand in the case of the least tightly fixing approach needs to cross small barriers of 12.1 kcal/mol for 1PYE, 12.5 kcal/mol for 2JFZ, 19.5 kcal/mol for 2YDV, 24.6 kcal/mol for 4JNJ and 8.2 kcal/mol for 1TSL. In contrast, all remarkable values of mode 1 and mode 2 are recorded at 99.3 and 37.2 kcal/mol for 1PYE, 147.2 and 35.4 kcal/mol for 2JFZ, 168.1 and 49.8 kcal/mol for 2YDV, 290.3 and 179.6 kcal/mol for 4JNJ, 45.4 and 15.7 kcal/mol for 1TSL. The mean curves of unbinding free energy from six systems are shown in Figs. S13–S18 of the SI file. When all free energy profiles are established it is easy to find that the unbinding barrier tends to decrease in the context of relaxing the protein.

### Flexibly restraining mode allows more residues to form contact with ligand

In order to better comprehend about how changes in protein structure could affect the ligand escape, we explore in this paragraph the interactions between proteins and ligands. Looking over 100 trajectories of a mode and 300 frames in each trajectory, a few steps are carried out: (1) we count the number of residues that make at least one contact with the ligand; full data are collected in Table S2(SI file), (2) we figure out the averaging number of hydrogen bonds in dependence on displacement, and (3) we plot the curve of the displacement-dependent interaction energy (IE).

**Figure 5:**
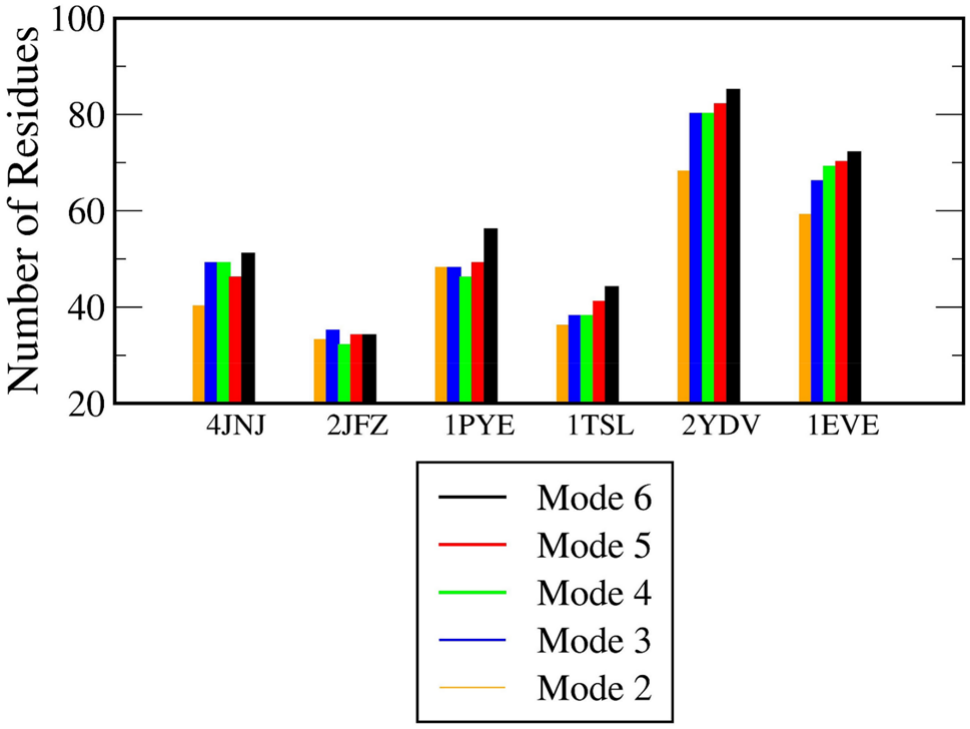
Number of residues that are formed at least with one contact with the ligand during its escape. Data are obtained over 100 independent trajectories. Data of mode 1 are not shown for the sake of clarity.

Interestingly, the flexibility of the protein is found to be diversified with respect to the protein–ligand interactive picture, because more residues are found to be coming into contact with the ligand during the dissociation process. Table S2 (SI file) shows the number of residues that have a time being in contact with the ligand. With 1EVE system, mode 1 results in only 55 residues that form contact with the ligand during the ligand exit. For mode 2 through mode 6, this quantity rises from 59, 66, 69, 70 to 72 residues. The proportional increase of this quantity of 1TSL and 2YDV systems is illustrated in Fig. 5.

**Figure 6:**
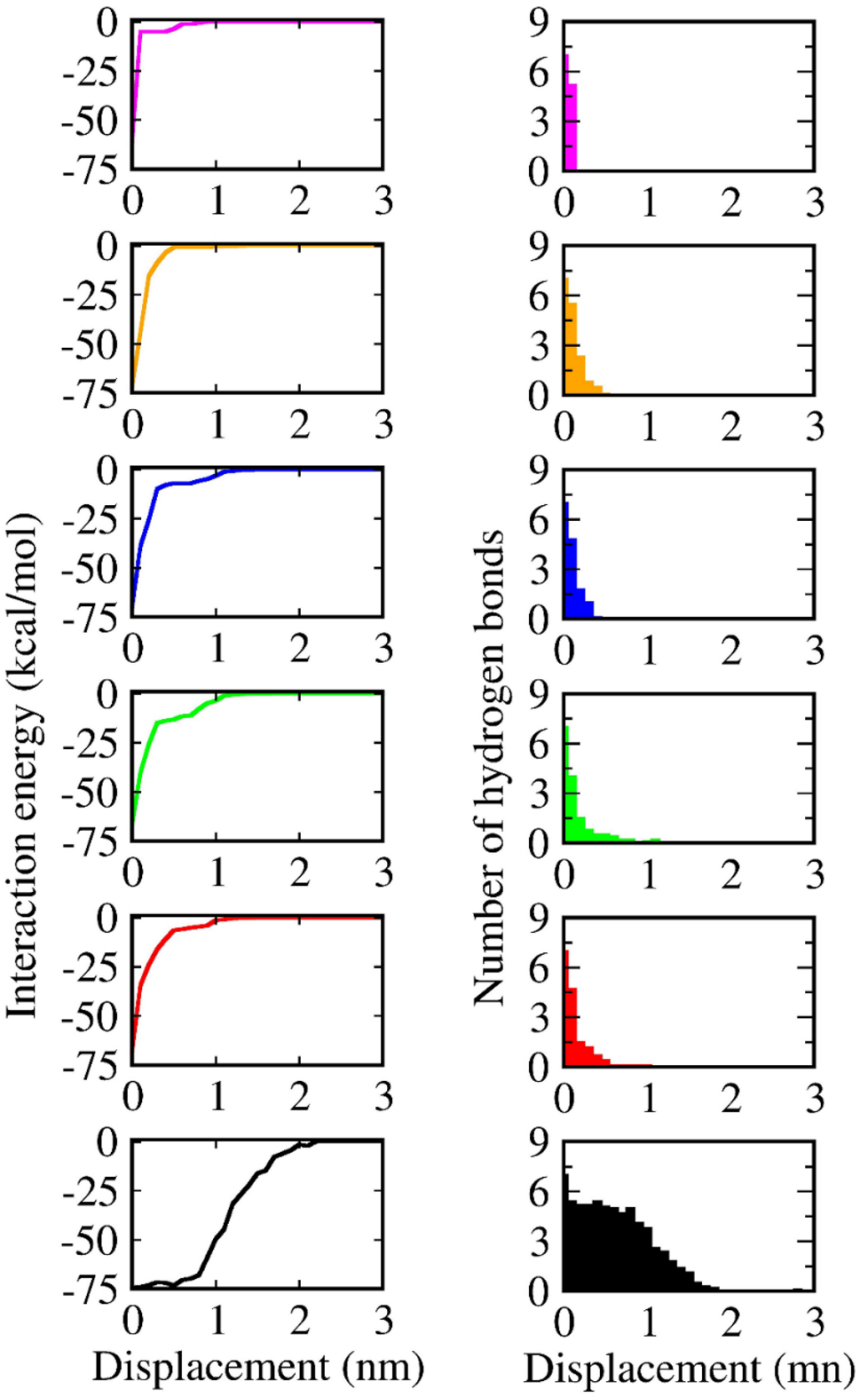
Averaging the interaction energy (left) and number of hydrogen bonds per frame (right) in the dependence of displacement obtained from the 4JNJ system under six restrained modes: mode 1 (in magenta), mode 2 (in orange), mode 3 (in blue), mode 4 (in green), mode 5 (in red), and mode 6 (in black).

In all complexes considered, numerical data indicates that the use of mode 1 and mode 2 tends to prevent the ability of the protein and narrow the space of configuration sampling. Notably, our displacement-dependent number of hydrogen bond in Fig. 6 and Figs. S20–S24 (SI file) demonstrates that this increase is mostly derived in the period of time after the rupture event occurs. That leads to a conclusion that when the protein is relaxed, it increases the fluctuation of residues in the nearby region. A simple fixing of the atoms allows as much as possible the residues to participate in the interaction with ligand. From the point of view of a natural event, a participation of more residues seems to encourage the ligand escaping. This observation needs to be explored with more evidence in the subsequent investigations.

### Flexibly restraining mode lengthens the ligand–protein harmonic potential

For a deeper analysis of the SMD simulations, we compute the interaction energy (IE) between both inhibitors and receptor, as a summation of polar (Coulomb) and non-polar (Van der Waals) interaction:

$${\text{IE}}={{\text{V}}}_{{\text{L}}-{\text{P}}}^{\text{VdW}}+{{\text{V}}}_{{\text{L}}-{\text{P}}}^{\text{Cou}}.$$

Each snapshot of saving data is submitted into one MD-step run for computing the ligand–protein interaction. Then the profile of non-bound interaction energy plotted within the dependence on ligand’s displacement is shown in Fig. 6. The full image of the interaction energies, the Coulomb potentials, and the Van der Waals potentials is shown in the SI file, from Figs. S7–S12. In particular, the length (L0) of the ligand–protein interaction potential is found to be changed as a result of the protein flexibility, which has ever been assumed to be constant in a previous theoretical study^60^. Notably, the interaction energy between the protein and ligand in the 4JNJ system is decreased into zero at the displacement of 0.5 nm (mode 2) and 2.0 nm (mode 6). This illustrates a significant difference from the smallest value of a narrower potential to the largest one of a wider potential. Since the length (L0) is an important parameter to construct the analytical expression of the protein–ligand harmonic potential, our collected data will make a reference to the building of the dependence of work on the pulling velocity in SMD study^76^.

## Concluding remarks

In summary, we have presented in the theoretical study six approaches for restraining the protein movement during a steered molecular dynamics simulation. Some mean values of rupture force and pulling work from neighboring modes, with the standard error added, are indistinguishable from each other. This is caused by the geometric features of the protein structure, where two neighboring modes are found to restrain quite similarly the groups of Cα atoms. The most important fact recognized in this scheme is that the 1st and 2nd standing positions, according to the largest values of $\langle {{\text{F}}}_{\text{max}}\rangle$ and $\langle {{\text{W}}}_{\text{pull}}\rangle$ obtained from modes 1 and 2, are clear-cut and invariant. This observation confirms that restrain of all heavy atoms or all Cα atoms cannot be considered as a good choice in applying steered molecular dynamics simulations.

Because significantly larger values have been generated in modes 1 and 2, it will create more difficulty when trying to come closer to an equilibrium dissociation. In contrast, if the protein is too flexible, the force will occasionally be applied primarily to stretching the protein structure rather than breaking the bonds between the ligand and amino acids. Unnecessary work could be additionally created. This leads to incorrect information and much challenge for the use of the pulling work method to determine the binding affinities between small molecules and macro proteins. More seriously, bulk water layer may push the protein rotating. Although it is rather hard to give a quantitative suggestion for every kind of protein, we would recommend a sufficiently suitable approach for applying in SMD simulations, that is *fixing all Cα atoms at a distance larger than 1.2 nm from the ligand, as we have applied in mode 3 and mode 4*.

Determination of a physical pathway for ligand release or entering is always a principle in molecular dynamic simulations. The evidence obtained in this study has raised a common status, that is, let protein move flexibly in such a way that the ligand can move out of the protein binding pocket. Lower unbinding energy barriers, lower pulling work, lower rupture force constitute a strong set of foundation for an easier escape of the ligand. In addition, a certain suspicion emerges, as to whether a protein, in the natural process when external force is absent, intentionally transform to release the ligand. We have reason to believe in the entrance or release process of the ligand, many intermediate structures of the protein impact more importantly than a functional conformation when protein has successfully folded. This issue is still not clear because of the limitation of protein-mediated conformations. Relatively little is actually known on the observed effect to reveal the role of mediated conformations.

### Supplementary Information

Supplementary Information 1.

Supplementary Information 2.

## Data Availability

Data including calculated results of SMD simulations are available in a pdf file while the input files are given in a zip file of the Electronic Supplementary Information (ESI).

## References

[1] Robinson AL (1986). Electron Microscope Inventors Share Nobel Physics Prize: Ernst Ruska built the first electron microscope in 1931; Gerd Binnig and Heinrich Rohrer developed the scanning tunneling microscope 50 years later. Science.

[2] Rugar D, Hansma P (1990). Atomic force microscopy. Phys. Today.

[3] Lo Giudice C, Dumitru AC, Alsteens D (2019). Probing ligand-receptor bonds in physiologically relevant conditions using AFM. Anal. Bioanal. Chem..

[4] Koehler M (2020). Control of ligand-binding specificity using photocleavable linkers in AFM force spectroscopy. Nano Lett..

[5] Fritz J, Katopodis AG, Kolbinger F, Anselmetti D (1998). Force-mediated kinetics of single P-selectin/ligand complexes observed by atomic force microscopy. Proc. Natl. Acad. Sci. U. S. A..

[6] Lemoine P, Dooley C, Morelli A, Harrison E, Dixon D (2022). AFM study of organic ligand packing on gold for nanoparticle drug delivery applications. Appl. Surf. Sci..

[7] Chowdhury N, Bagchi A (2022). A drug repurposing endeavor to discover a multi-targeting ligand against RhlR and LasR proteins from opportunistic human pathogen Pseudomonas aeruginosa. J. Mol. Model..

[8] Legittimo F, Marini M, Stassi S, Di Fabrizio E, Ricciardi C (2023). Real-time monitoring of temperature-dependent structural transitions in DNA nanomechanical resonators: Unveiling the DNA–ligand interactions for biomedical applications. ACS Appl. Nano Mater..

[9] Copeland RA, Pompliano DL, Meek TD (2006). Drug-target residence time and its implications for lead optimization. Nat. Rev. Drug Discov..

[10] Nunez S, Venhorst J, Kruse CG (2012). Target-drug interactions: First principles and their application to drug discovery. Drug Discov. Today.

[11] Shamir M, Bar-On Y, Phillips R, Milo R (2016). SnapShot: Timescales in cell biology. Cell.

[12] Bryant, R., Katz, R. H. & Lazowska, E. D. (December, 2008).

[13] Klco N, Roggero A, Savage MJ (2022). Standard model physics and the digital quantum revolution: Thoughts about the interface. Rep. Prog. Phys..

[14] Grubmuller H, Heymann B, Tavan P (1996). Ligand binding: Molecular mechanics calculation of the streptavidin-biotin rupture force. Science.

[15] Evans E, Ritchie K (1997). Dynamic strength of molecular adhesion bonds. Biophys. J..

[16] Isralewitz B, Izrailev S, Schulten K (1997). Binding pathway of retinal to bacterio-opsin: A prediction by molecular dynamics simulations. Biophys. J..

[17] Izrailev S, Stepaniants S, Balsera M, Oono Y, Schulten K (1997). Molecular dynamics study of unbinding of the avidin-biotin complex. Biophys. J..

[18] Rief M, Gautel M, Oesterhelt F, Fernandez JM, Gaub HE (1997). Reversible unfolding of individual titin immunoglobulin domains by AFM. Science.

[19] Heymann B, Grubmüller H (1999). AN02/DNP-hapten unbinding forces studied by molecular dynamics atomic force microscopy simulations. Chem. Phys. Lett..

[20] Chovancova E (2012). CAVER 3.0: A tool for the analysis of transport pathways in dynamic protein structures. PLoS Comput. Biol..

[21] Chwastyk M (2014). Theoretical tests of the mechanical protection strategy in protein nanomechanics. Proteins.

[22] Zhao Y, Chwastyk M, Cieplak M (2017). Structural entanglements in protein complexes. J. Chem. Phys..

[23] Nguyen HL, Thai NQ, Truong DT, Li MS (2020). Remdesivir strongly binds to both RNA-dependent RNA polymerase and main protease of SARS-CoV-2: Evidence from molecular simulations. J. Phys. Chem. B.

[24] Truong DT, Li MS (2018). Probing the binding affinity by Jarzynski's nonequilibrium binding free energy and rupture time. J. Phys. Chem. B.

[25] Gunnoo M (2018). Steered molecular dynamics simulations reveal the role of Ca(2+) in regulating mechanostability of cellulose-binding proteins. Phys. Chem. Chem. Phys..

[26] Chwastyk M, Bernaola AP, Cieplak M (2015). Statistical radii associated with amino acids to determine the contact map: fixing the structure of a type I cohesin domain in the Clostridium thermocellum cellulosome. Phys. Biol..

[27] Zhao Y, Chwastyk M, Cieplak M (2017). Topological transformations in proteins: Effects of heating and proximity of an interface. Sci. Rep..

[28] Li MS (2017). Ligand migration and steered molecular dynamics in drug discovery: Comment on "Ligand diffusion in proteins via enhanced sampling in molecular dynamics" by Jakub Rydzewski and Wieslaw Nowak. Phys. Life Rev..

[29] Iida S, Tomoshi K (2022). Free energy and kinetic rate calculation via non-equilibrium molecular simulation: Application to biomolecules. Biophys. Rev..

[30] Do PC, Lee EH, Le L (2018). Steered molecular dynamics simulation in rational drug design. J. Chem. Inf. Model..

[31] Kosztin D, Izrailev S, Schulten K (1999). Unbinding of retinoic acid from its receptor studied by steered molecular dynamics. Biophys. J..

[32] Le L, Lee EH, Hardy DJ, Truong TN, Schulten K (2010). Molecular dynamics simulations suggest that electrostatic funnel directs binding of Tamiflu to influenza N1 neuraminidases. PLoS Comput. Biol..

[33] Wriggers W, Schulten K (1999). Investigating a back door mechanism of actin phosphate release by steered molecular dynamics. Proteins.

[34] Suan Li M, Khanh Mai B (2012). Steered molecular dynamics-a promising tool for drug design. Curr. Bioinform..

[35] Mai BK, Li MS (2011). Neuraminidase inhibitor R-125489–a promising drug for treating influenza virus: Steered molecular dynamics approach. Biochem. Biophys. Res. Commun..

[36] Nicolini P, Frezzato D, Gellini C, Bizzarri M, Chelli R (2013). Toward quantitative estimates of binding affinities for protein-ligand systems involving large inhibitor compounds: A steered molecular dynamics simulation route. J. Comput. Chem..

[37] Xu Y (2003). How does huperzine A enter and leave the binding gorge of acetylcholinesterase? Steered molecular dynamics simulations. J. Am. Chem. Soc..

[38] Shen L (2003). Steered molecular dynamics simulation on the binding of NNRTI to HIV-1 RT. Biophys. J..

[39] Niu C (2005). Dynamic mechanism of E2020 binding to acetylcholinesterase: A steered molecular dynamics simulation. J. Phys. Chem. B.

[40] Zhang D, Gullingsrud J, McCammon JA (2006). Potentials of mean force for acetylcholine unbinding from the alpha7 nicotinic acetylcholine receptor ligand-binding domain. J. Am. Chem. Soc..

[41] Vuong QV, Nguyen TT, Li MS (2015). A new method for navigating optimal direction for pulling ligand from binding pocket: Application to ranking binding affinity by steered molecular dynamics. J. Chem. Inf. Model..

[42] Chen LY (2015). Hybrid steered molecular dynamics approach to computing absolute binding free energy of ligand-protein complexes: A brute force approach that is fast and accurate. J. Chem. Theory Comput..

[43] Potterton A (2019). Ensemble-based steered molecular dynamics predicts relative residence time of A(2A) receptor binders. J. Chem. Theory Comput..

[44] Wolf S, Lickert B, Bray S, Stock G (2020). Multisecond ligand dissociation dynamics from atomistic simulations. Nat. Commun..

[45] Paul F, Thomas T, Roux B (2020). Diversity of long-lived intermediates along the binding pathway of imatinib to abl kinase revealed by MD simulations. J. Chem. Theory Comput..

[46] Trezza A, Iovinelli D, Santucci A, Prischi F, Spiga O (2020). An integrated drug repurposing strategy for the rapid identification of potential SARS-CoV-2 viral inhibitors. Sci. Rep..

[47] Cao DT (2021). Molecular design of anticancer drugs from marine fungi derivatives. RSC Adv..

[48] Awad IE, Abu-Saleh AAA, Sharma S, Yadav A, Poirier RA (2022). High-throughput virtual screening of drug databanks for potential inhibitors of SARS-CoV-2 spike glycoprotein. J. Biomol. Struct. Dyn..

[49] Sedighpour D, Taghizadeh H (2022). The effects of mutation on the drug binding affinity of Neuraminidase: Case study of Capsaicin using steered molecular dynamics simulation. J. Mol. Model..

[50] Davis AM, Teague SJ (1999). Hydrogen bonding, hydrophobic interactions, and failure of the rigid receptor hypothesis. Angew Chem. Int. Ed. Engl..

[51] Carlson HA (2002). Protein flexibility is an important component of structure-based drug discovery. Curr. Pharm. Des..

[52] Adcock SA, McCammon JA (2006). Molecular dynamics: Survey of methods for simulating the activity of proteins. Chem. Rev..

[53] Teague SJ (2003). Implications of protein flexibility for drug discovery. Nat. Rev. Drug Discov..

[54] Amaral M (2017). Protein conformational flexibility modulates kinetics and thermodynamics of drug binding. Nat. Commun..

[55] Stank A, Kokh DB, Fuller JC, Wade RC (2016). Protein binding pocket dynamics. Acc Chem. Res..

[56] Fang Y (2019). Catalytic reactions within the cavity of coordination cages. Chem. Soc. Rev..

[57] Kokkonen P, Bednar D, Pinto G, Prokop Z, Damborsky J (2019). Engineering enzyme access tunnels. Biotechnol. Adv..

[58] Zhang Z (2016). Steered molecular dynamics study of inhibitor binding in the internal binding site in dehaloperoxidase-hemoglobin. Biophys. Chem..

[59] de Aquino BRH, Chwastyk M, Mioduszewski Ł, Cieplak M (2020). Networks of interbasin traffic in intrinsically disordered proteins. Phys. Rev. Res..

[60] Mioduszewski Ł, Bednarz J, Chwastyk M, Cieplak M (2023). Contact-based molecular dynamics of structured and disordered proteins in a coarse-grained model: Fixed contacts, switchable contacts and those described by pseudo-improper-dihedral angles. Comput. Phys. Commun..

[61] Chwastyk M, Jaskolski M, Cieplak M (2014). Structure-based analysis of thermodynamic and mechanical properties of cavity-containing proteins–Case study of plant pathogenesis-related proteins of class 10. FEBS J..

[62] Chwastyk M, Jaskolski M, Cieplak M (2016). The volume of cavities in proteins and virus capsids. Proteins.

[63] Chwastyk M, Panek EA, Malinowski J, Jaskolski M, Cieplak M (2020). Properties of cavities in biological structures-A survey of the protein data bank. Front. Mol. Biosci..

[64] Pall S (2020). Heterogeneous parallelization and acceleration of molecular dynamics simulations in GROMACS. J. Chem. Phys..

[65] The PyMOL molecular graphics system. Retrieved from http://www.pymol.org/pymol (2020).

[66] DeLano WL (2002). Pymol: An open-source molecular graphics tool. CCP4 Newslett. Protein Crystallogr..

[67] Gaussian 16 Rev. C.01 (Wallingford, CT, 2016).

[68] Fox T, Kollman PA (1998). Application of the RESP methodology in the parametrization of organic solvents. J. Phys. Chem. B.

[69] Wang J, Wolf RM, Caldwell JW, Kollman PA, Case DA (2004). Development and testing of a general amber force field. J. Comput. Chem..

[70] Sousa da Silva AW, Vranken WF (2012). ACPYPE—AnteChamber PYthon Parser interfacE. BMC Res. Notes.

[71] Darden T, York D, Pedersen L (1993). Particle mesh Ewald: An N⋅log(N) method for Ewald sums in large systems. J. Chem. Phys..

[72] Andersen HC (1983). Rattle: A “velocity” version of the shake algorithm for molecular dynamics calculations. J. Comput. Phys..

[73] Stourac J (2019). Caver Web 1.0: Identification of tunnels and channels in proteins and analysis of ligand transport. Nucleic Acids Res..

[74] Ho K, Truong DT, Li MS (2020). How good is Jarzynski's equality for computer-aided drug design?. J. Phys. Chem. B.

[75] Dudko OK, Hummer G, Szabo A (2006). Intrinsic rates and activation free energies from single-molecule pulling experiments. Phys. Rev. Lett..

[76] Pham HA, Truong DT, Li MS (2021). Dependence of work on the pulling speed in mechanical ligand unbinding. J. Phys. Chem. B.

