# Supplementary material for: Treatment of flexibility of protein backbone in simulations of protein–ligand interactions using steered molecular dynamics
Source: Sci Rep. 2024 May 7;14:10475. doi: 10.1038/s41598-024-59899-3 (PMC11076533; doi:10.1038/s41598-024-59899-3)
Supplement: Supplementary file 2 — Supplementary Information 2. [file 41598_2024_59899_MOESM2_ESM.pdf]

Electronic Supplementary Information (ESI)

Treatment of flexibility of protein backbone in simulations of protein-ligand interactions using steered molecular dynamics

Duc Toan Truong,[1,2] Kiet Ho,[3] Pham Dinh Quoc Huy,[4] Mateusz Chwastyk,[4] Thai Nguyen-Minh,[5] Minh Tho Nguyen[1,2,*]

[1] Laboratory for Chemical Computation and Modeling, Institute for Computational Science and Artificial Intelligence, Van Lang University, Ho Chi Minh City, 70000 Vietnam

Email: minhtho.nguyen@vlu.edu.vn

[2] Faculty of Applied Technology, School of Technology, Van Lang University, Ho Chi Minh City, 70000 Vietnam

[3] Institute for Computational Science and Technology (ICST), Quang Trung Software City, Ho Chi Minh City, 70000 Vietnam

[4] Institute of Physics, Polish Academy of Sciences, Warsaw, Poland

[5] University of Medicine and Pharmacy at Ho Chi Minh City, 70000 Vietnam

Input data:

Each input file contains thousands of lines going up to ~10 MB size. With the 6x6 systems considered, input files are relatively large to be given in the ESI file. Interested readers can contact us for these input data.

***Table S1**: Number of hydrogen bonds the protein and ligand have formed per one dissociation process. Each pulling trajectory was saved into 300 snapshots. Data was averaged from 100 independence trajectories, six modes in six systems.*

(a)	***Averaging number of hydrogen bonds per trajectory in different restrained methods***					
**PDB-ID**	**4JNJ**	**2JFZ**	**1PYE**	**1TSL**	**2YDV**	**1EVE**
Mode 1	73.2	39.9	303	121.2	143.1	12.99
Mode 2	97.2	14.01	71.1	137.1	198.9	12
Mode 3	88.2	42.9	106.8	102.9	204.9	6.99
Mode 4	92.4	35.1	88.8	51.3	215.2	15
Mode 5	96	36.9	67.8	116.7	229.7	9.99
Mode 6	399	24.99	112.2	99.6	267	27.99

Table S2: Number of amino acids that have formed one contact with ligand.

Data obtained from 100 independence trajectories, six modes in six systems.

		Number of amino aacid				
**PDB-ID**	**4JNJ**	**2JFZ**	**1PYE**	**1TSL**	**2YDV**	**1EVE**
Mode 1	39	30	44	30	62	55
Mode 2	40	33	48	36	68	59
Mode 3	49	35	48	38	80	66
Mode 4	49	32	46	38	80	69
Mode 5	46	34	49	41	82	70
Mode 6	51	34	56	44	85	72

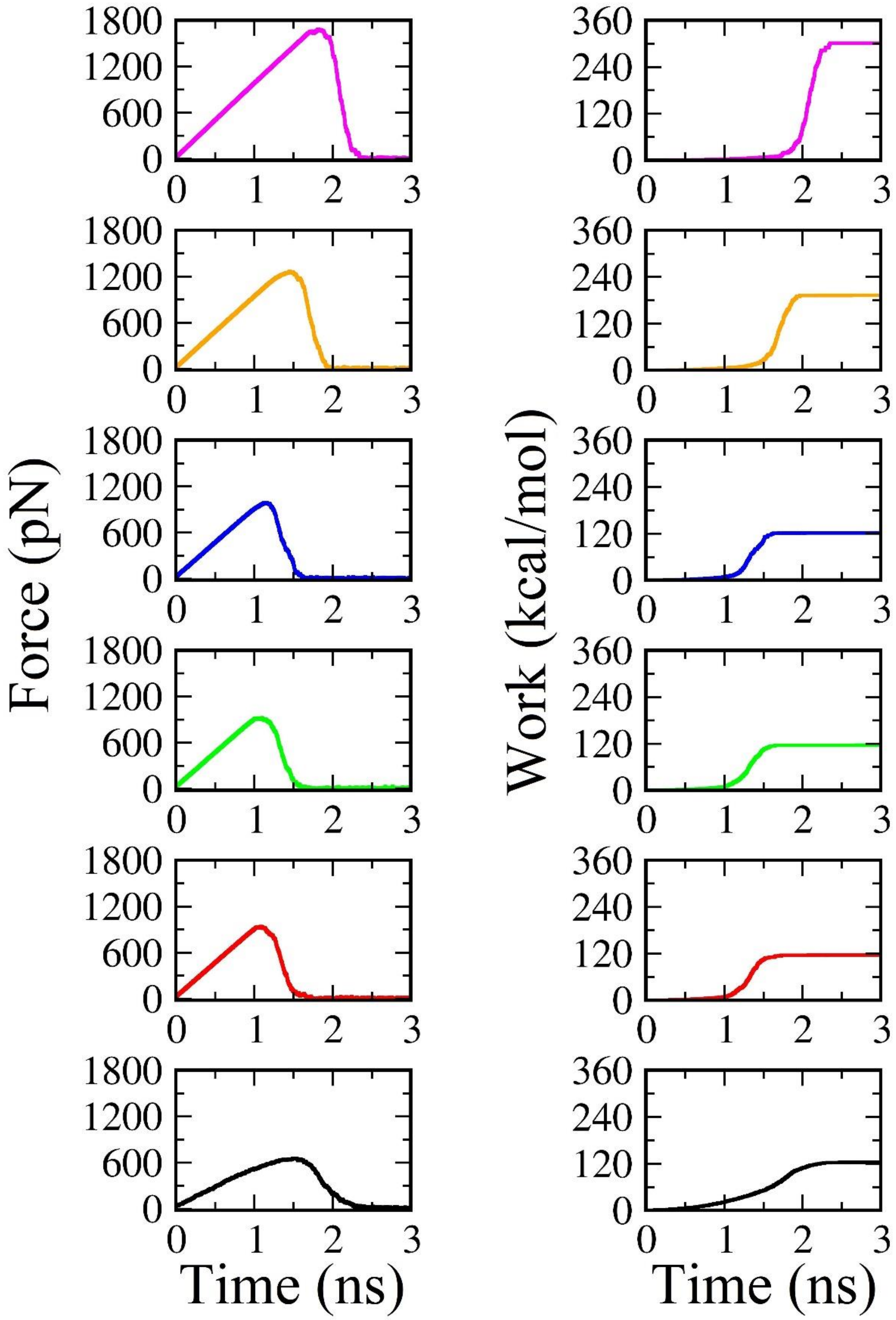

Figure S1: The time – dependent force and the time – dependent work averaged from 100 independent trajectories of 4JNJ system. Data from six different restraining modes are shown in magenta (mode 1), orange (mode 2), blue (mode 3), green (mode 4), red (mode 5) and black (mode 6).

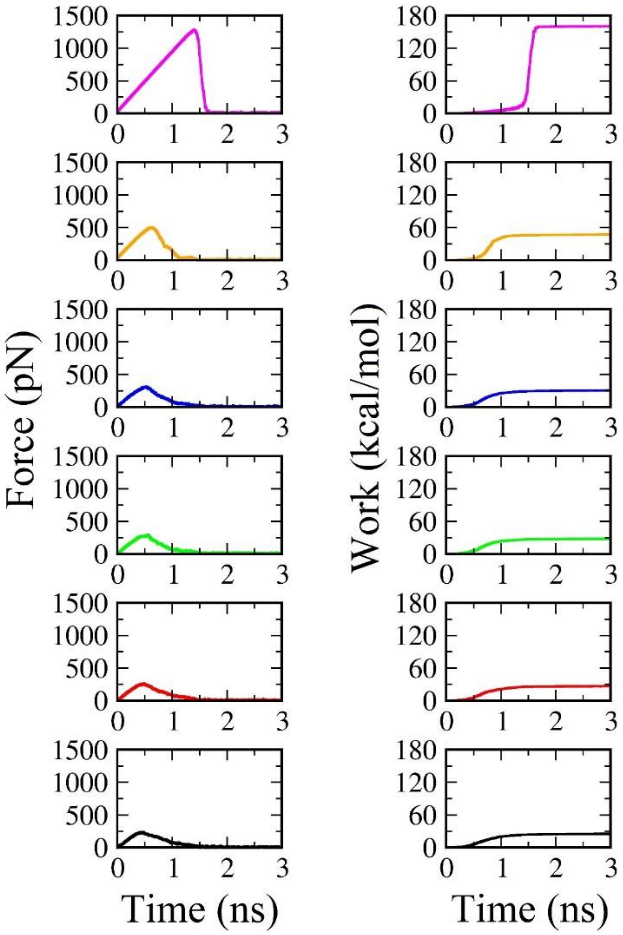

Figure S2: The time – dependent force and the time – dependent work averaged from 100 independent trajectories of 2JFZ. Data from six different restraining modes are shown in magenta (mode 1), orange (mode 2), blue (mode 3), green (mode 4), red (mode 5) and black (mode 6).

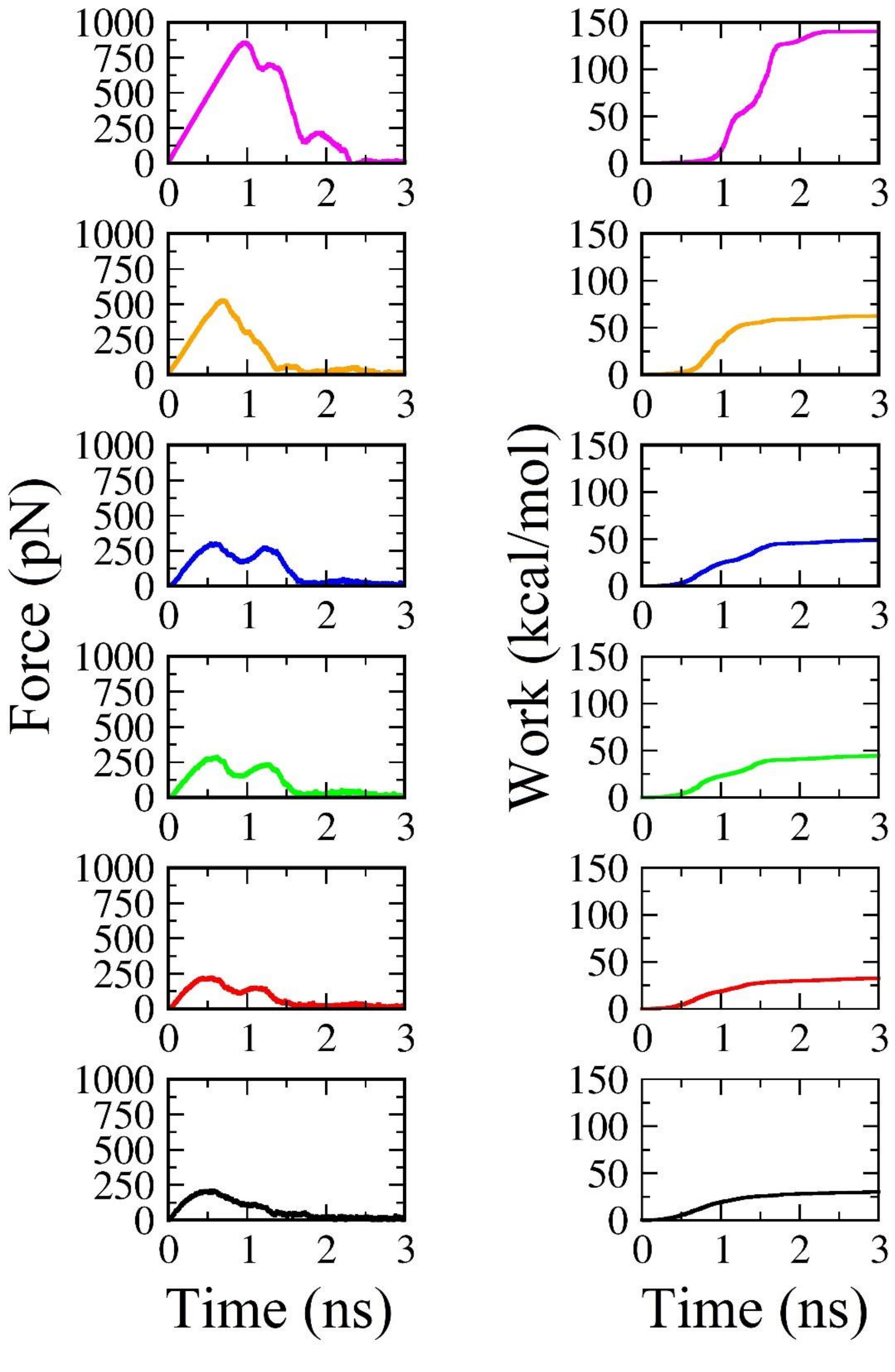

Figure S3: The time – dependent force and the time – dependent work averaged from 100 independent trajectories of 1PYE. Data from six different restraining modes are shown in magenta (mode 1), orange (mode 2), blue (mode 3), green (mode 4), red (mode 5) and black (mode 6).

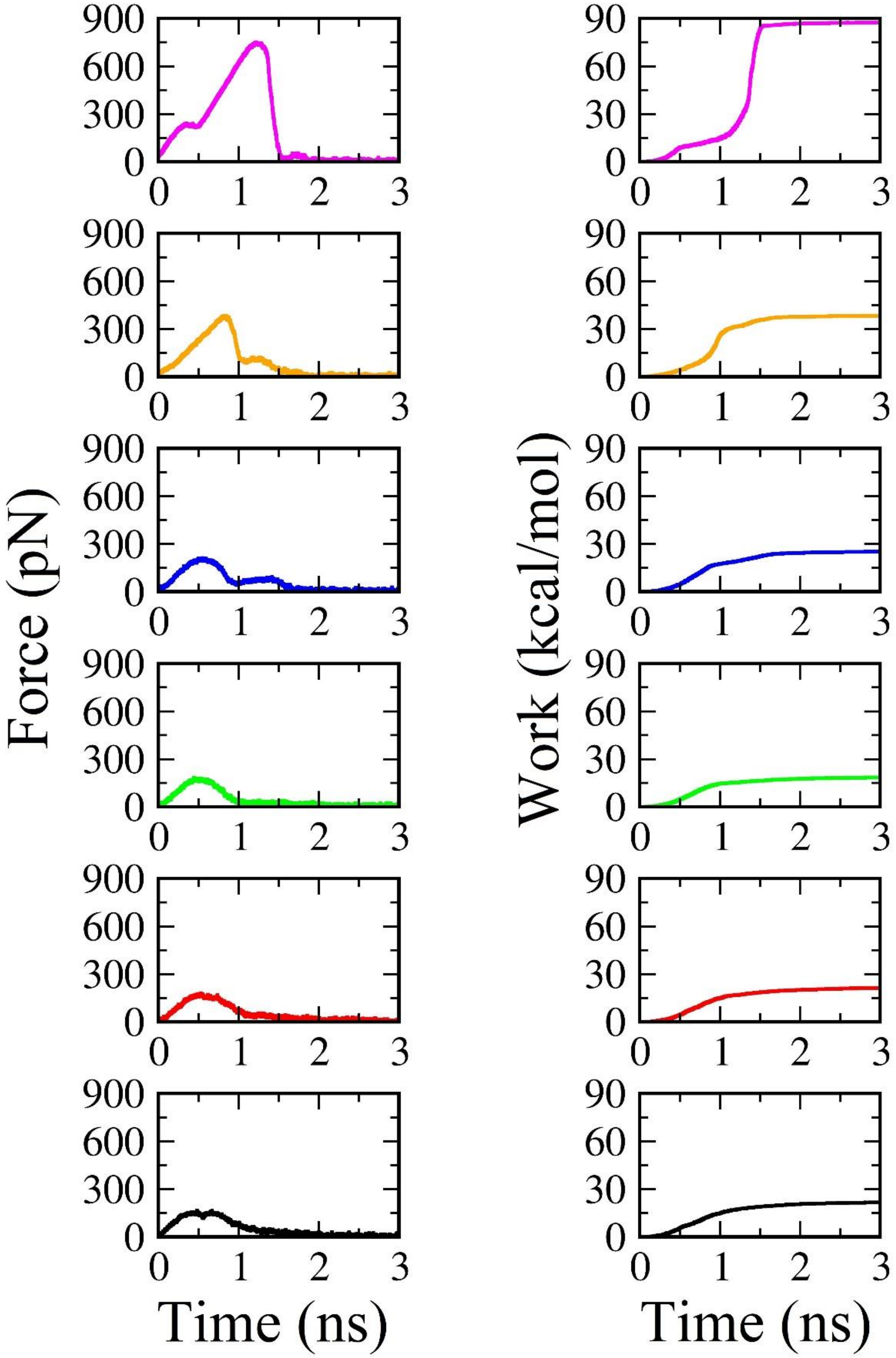

Figure S4: The time – dependent force and the time – dependent work averaged from 100 independent trajectories of 1TSL. Data from six different restraining modes are shown in magenta (mode 1), orange (mode 2), blue (mode 3), green (mode 4), red (mode 5) and black (mode 6).

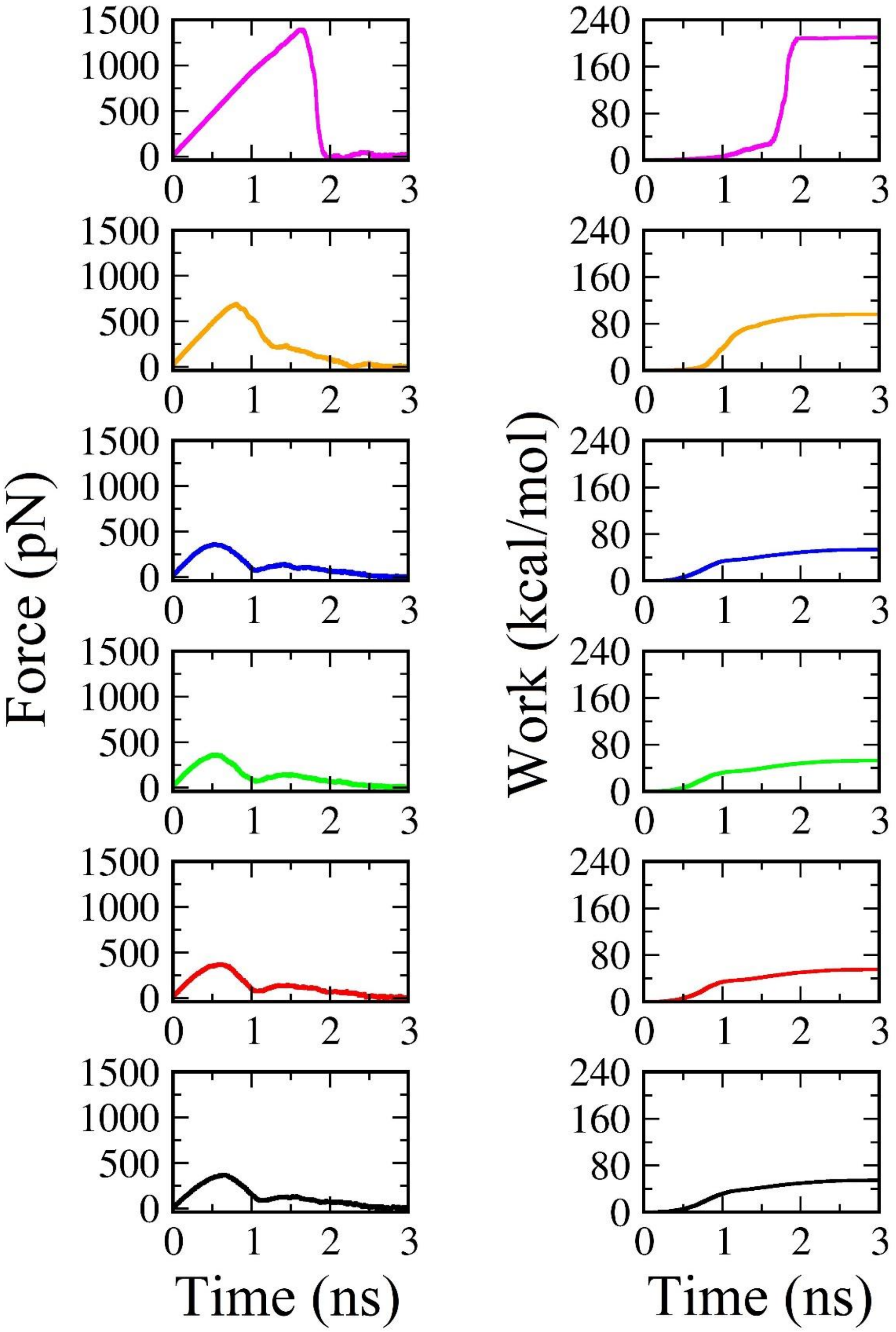

Figure S5: The time – dependent force and the time – dependent work averaged from 100 independent trajectories of 1YDV. Data from six different restraining modes are shown in magenta (mode 1), orange (mode 2), blue (mode 3), green (mode 4), red (mode 5) and black (mode 6).

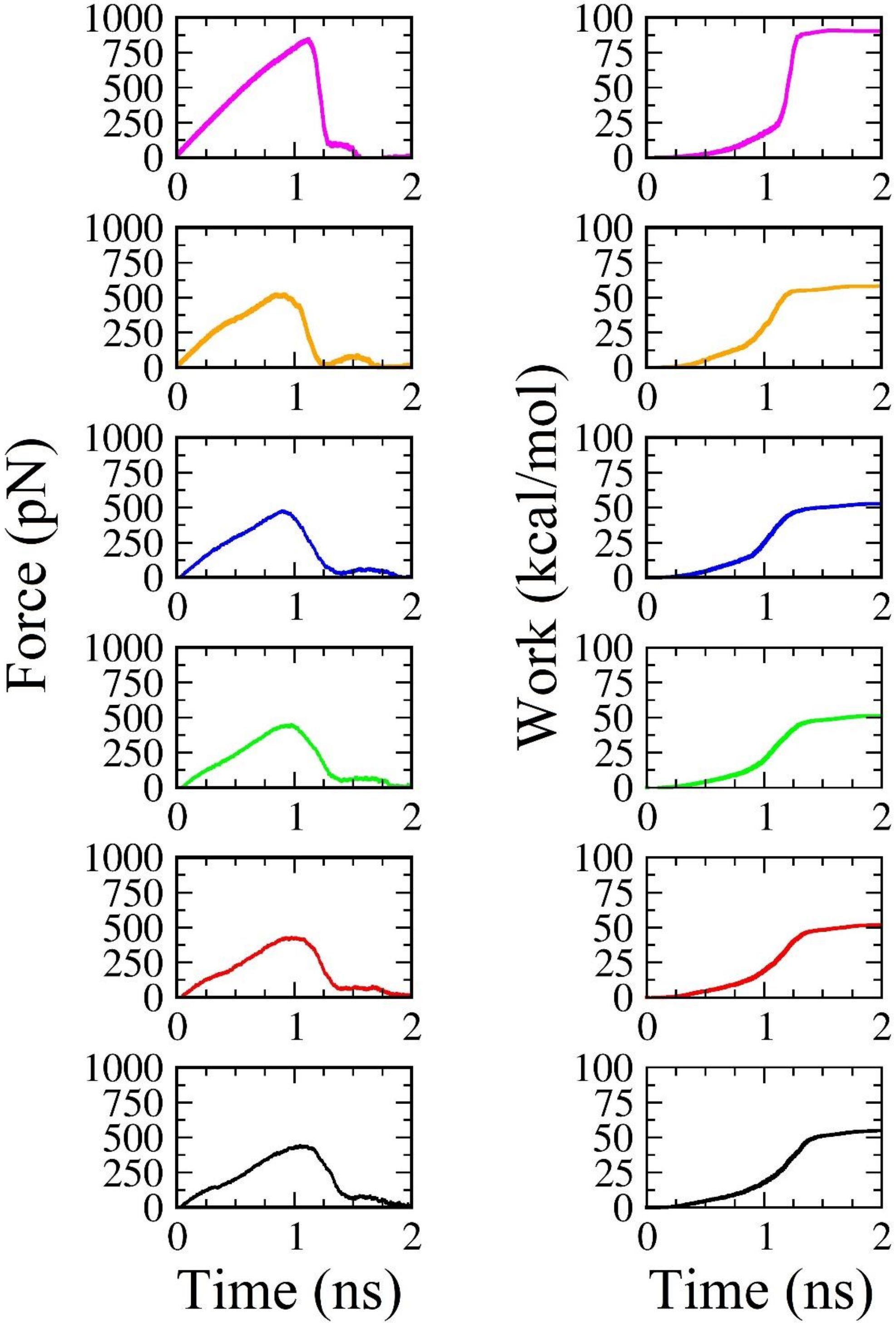

Figure S6: The time – dependent force and the time – dependent work averaged from 100 independent trajectories of 1EVE. Data from six different restraining modes are shown in magenta (mode 1), orange (mode 2), blue (mode 3), green (mode 4), red (mode 5) and black (mode 6).

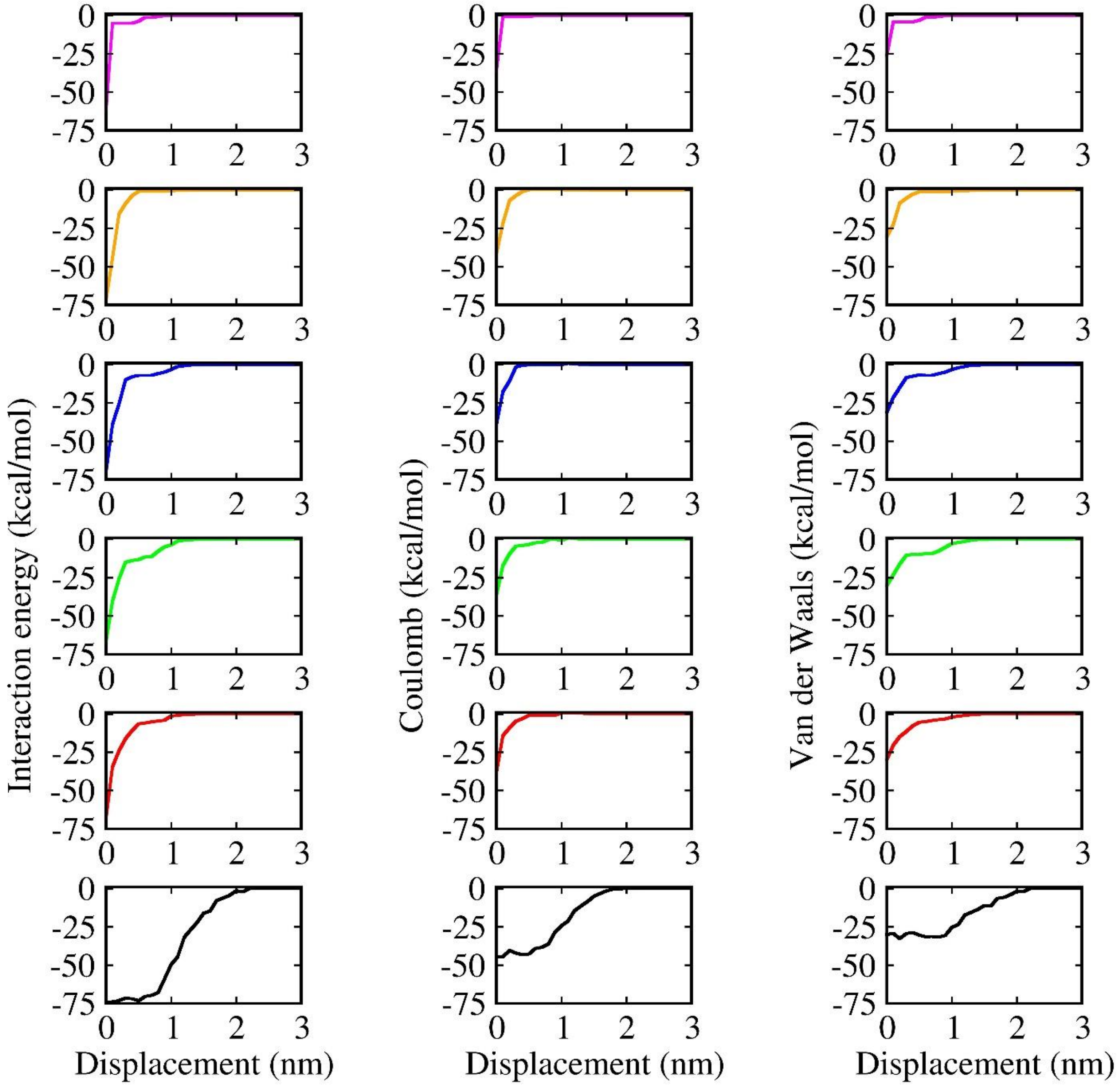

Figure S7: Data obtained from 6 restrained modes: mode 1 (in magenta), mode 2 (in orange), mode 3 (in blue), mode 4 (in green), mode 5 (in red) and mode 6 (in black). The LJ potential energy (right), the electrostatic potential energy (center) and the total interaction energy (left) between ligand and protein in case of 4JNJ system.

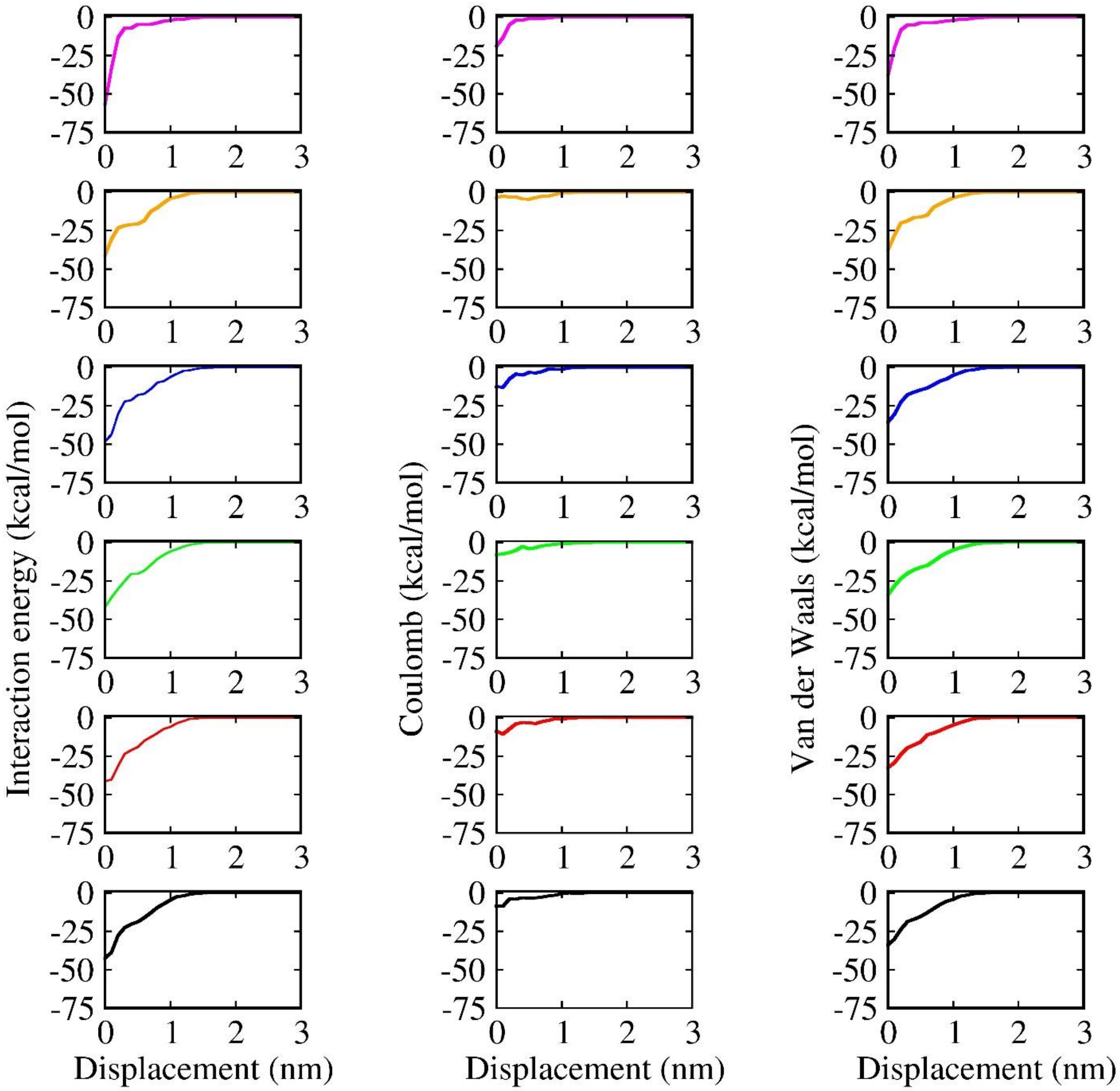

Figure S8: Data obtained from 6 restrained modes: mode 1 (in magenta), mode 2 (in orange), mode 3 (in blue), mode 4 (in green), mode 5 (in red) and mode 6 (in black). The LJ potential energy (right), the electrostatic potential energy (center) and the total interaction energy (left) between ligand and protein in case of 2JFZ system.

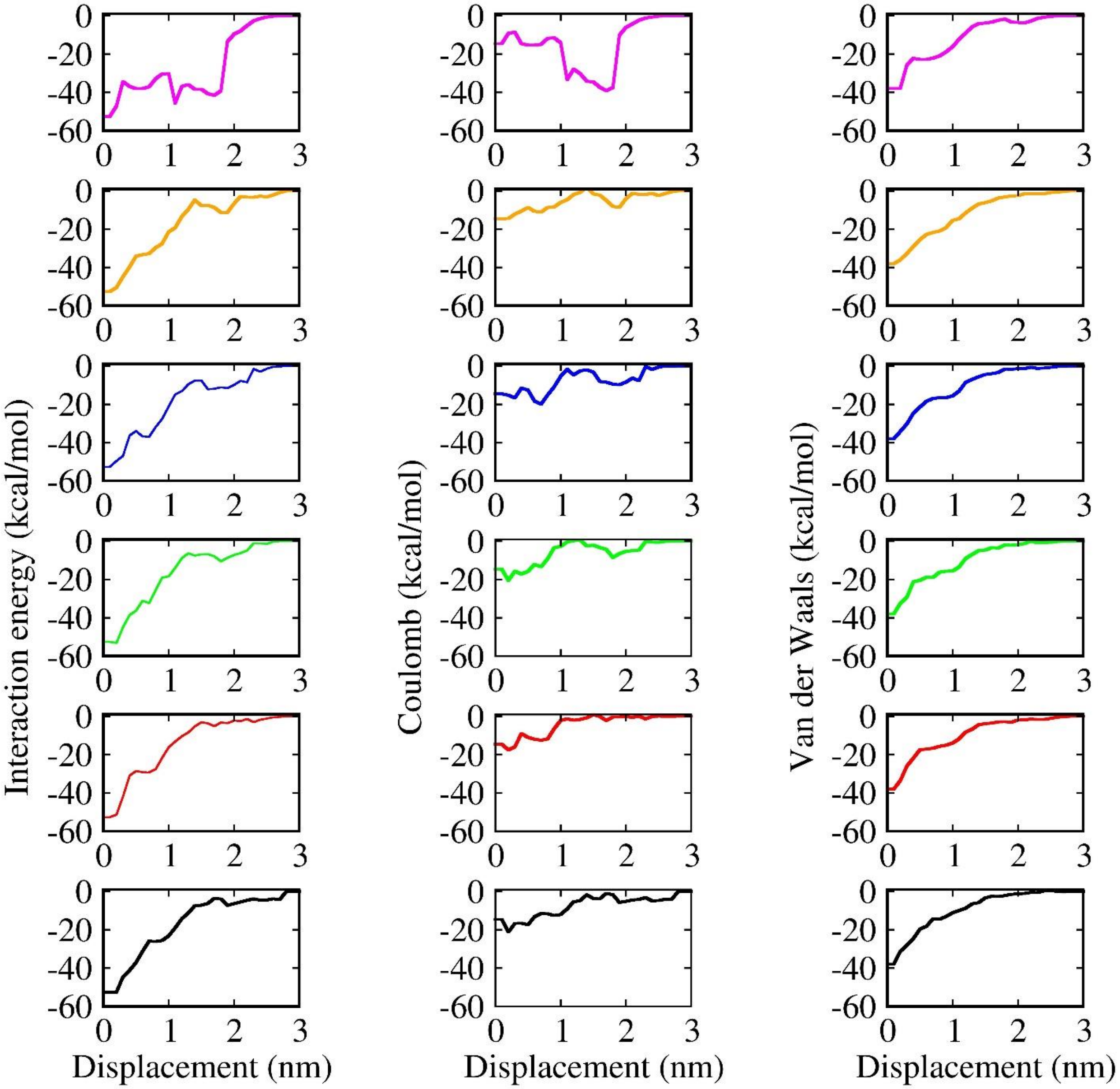

Figure S9: Data obtained from 6 restrained modes: mode 1 (in magenta), mode 2 (in orange), mode 3 (in blue), mode 4 (in green), mode 5 (in red) and mode 6 (in black). The LJ potential energy (right), the electrostatic potential energy (center) and the total interaction energy (left) between ligand and protein in case of 1PYE system.

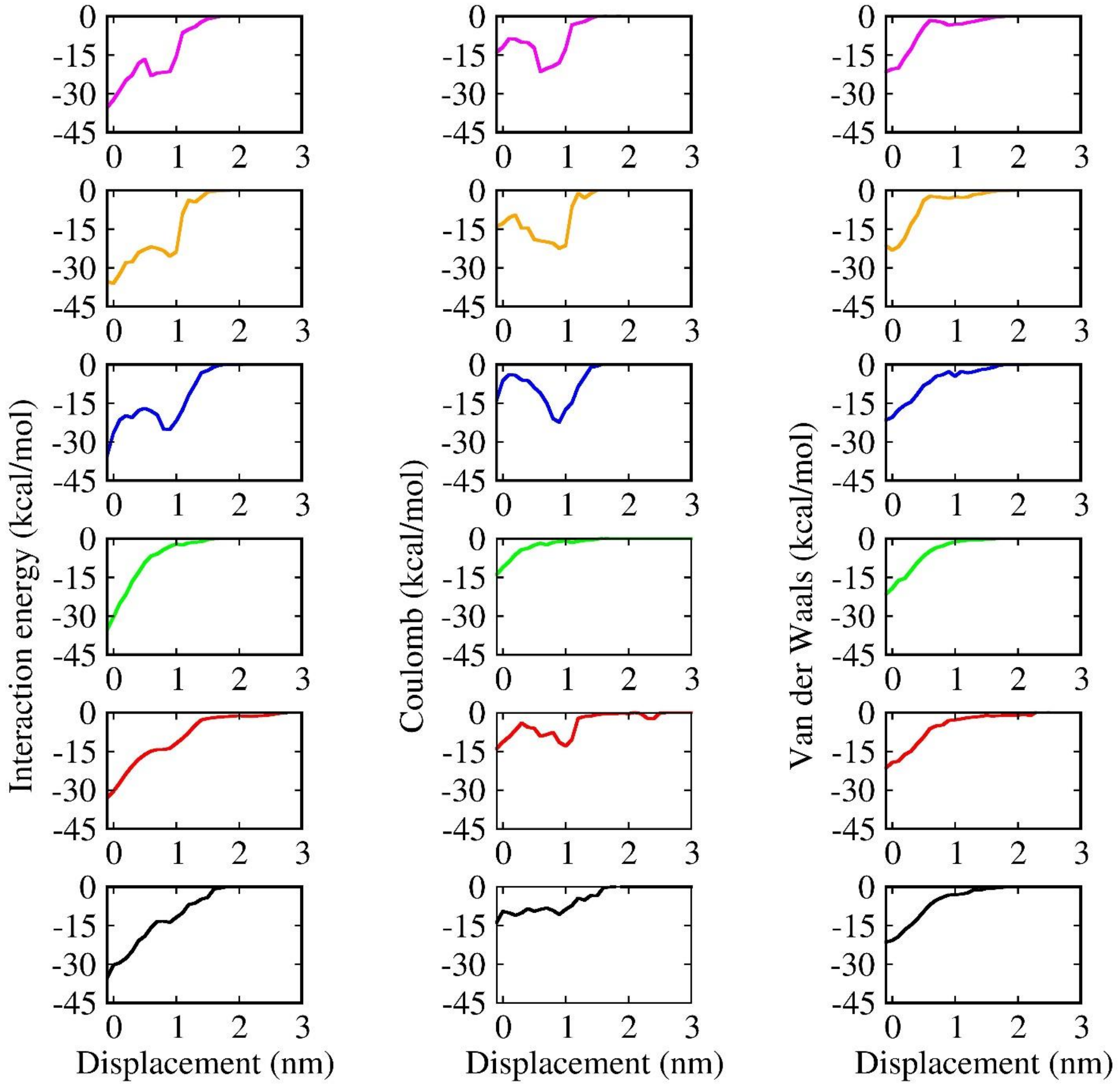

Figure S10: Data obtained from 6 restrained modes: mode 1 (in magenta), mode 2 (in orange), mode 3 (in blue), mode 4 (in green), mode 5 (in red) and mode 6 (in black). The LJ potential energy (right), the electrostatic potential energy (center) and the total interaction energy (left) between ligand and protein in case of 1TSL system.

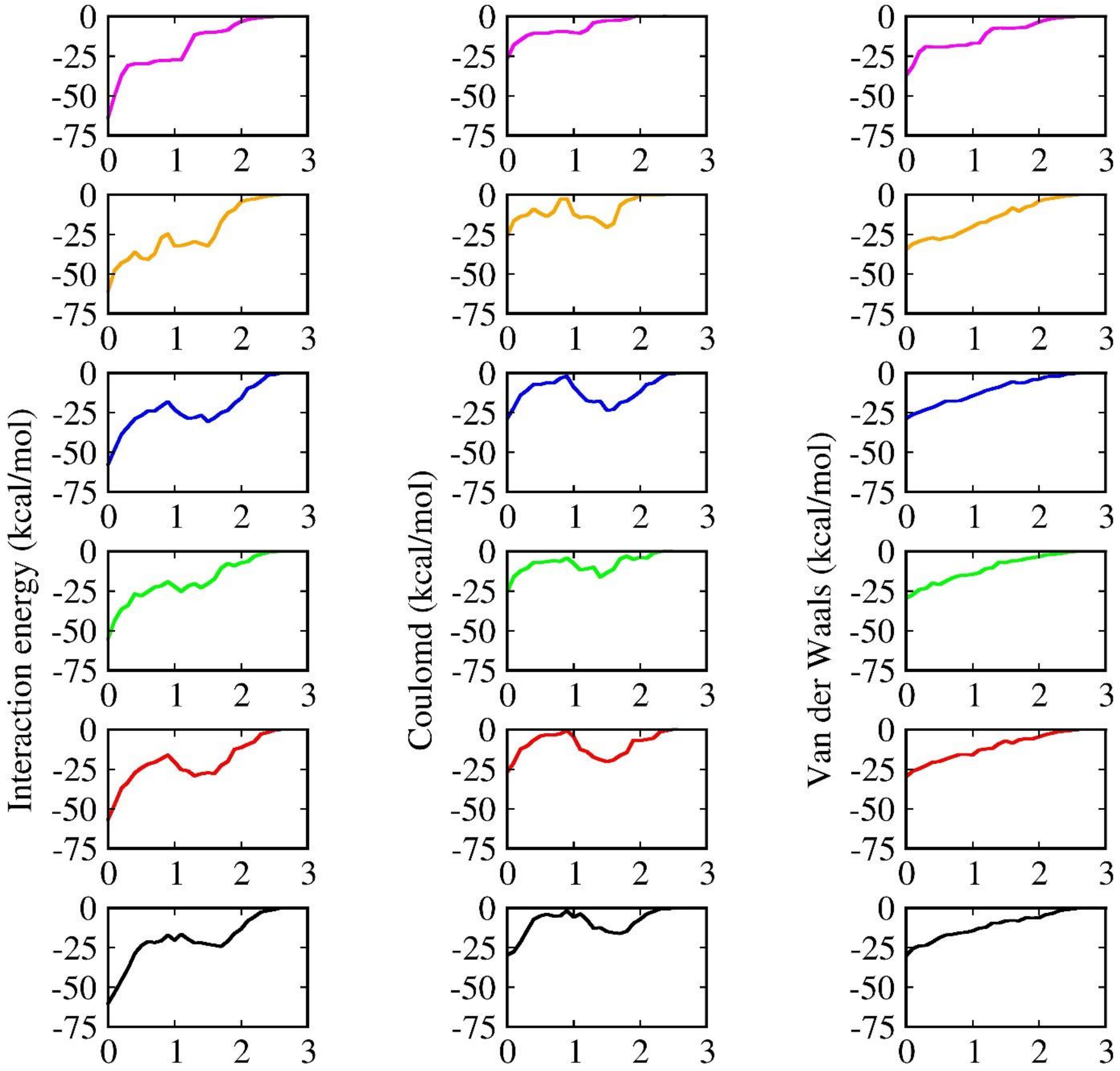

Figure S11: Data obtained from 6 restrained modes: mode 1 (in magenta), mode 2 (in orange), mode 3 (in blue), mode 4 (in green), mode 5 (in red) and mode 6 (in black). The LJ potential energy (right), the electrostatic potential energy (center) and the total interaction energy (left) between ligand and protein in case of 2YDV system.

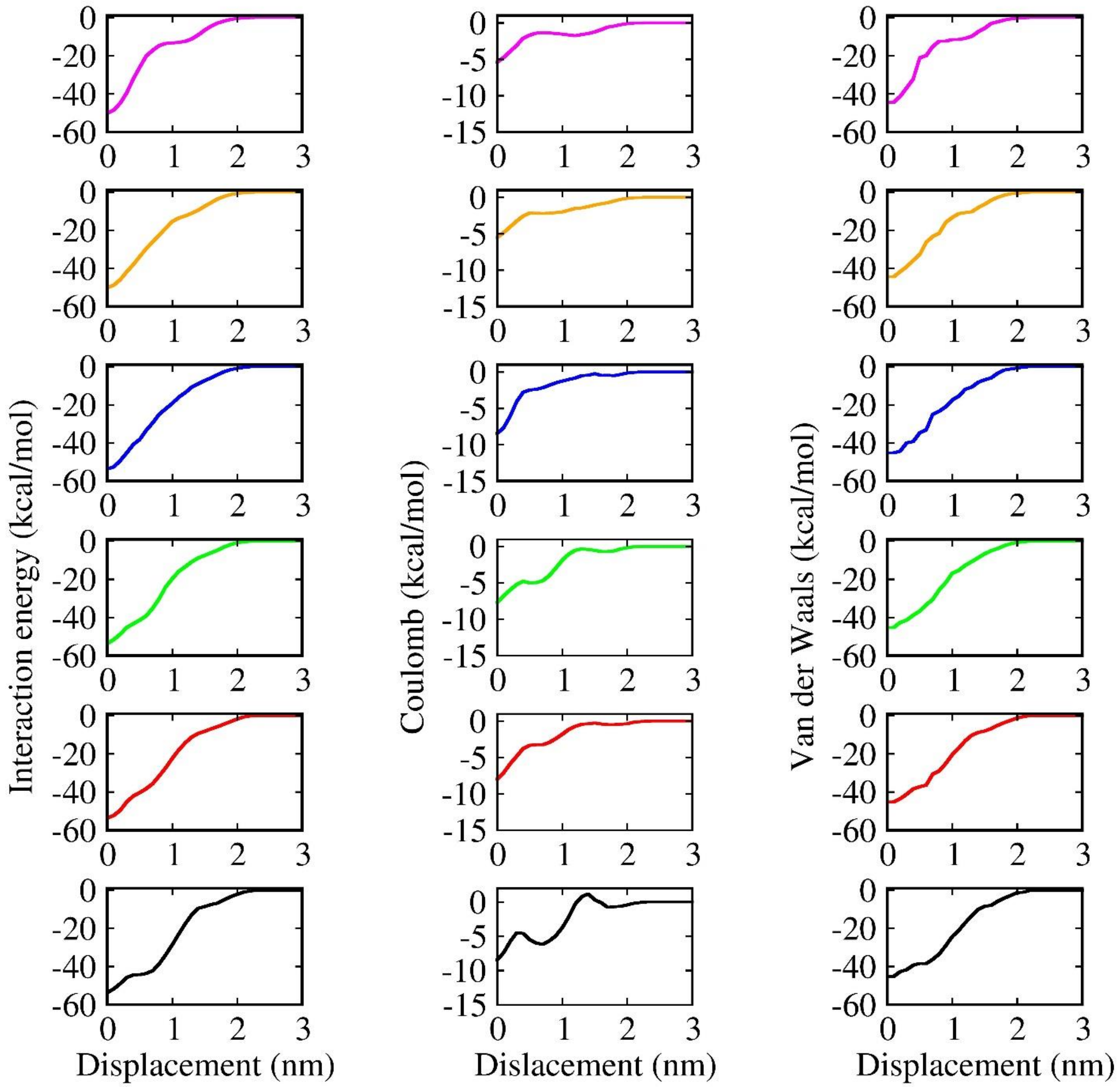

Figure S12: Data obtained from 6 restrained modes: mode 1 (in magenta), mode 2 (in orange), mode 3 (in blue), mode 4 (in green), mode 5 (in red) and mode 6 (in black). The LJ potential energy (right), the electrostatic potential energy (center) and the total interaction energy (left) between ligand and protein in case of 1EVE system.

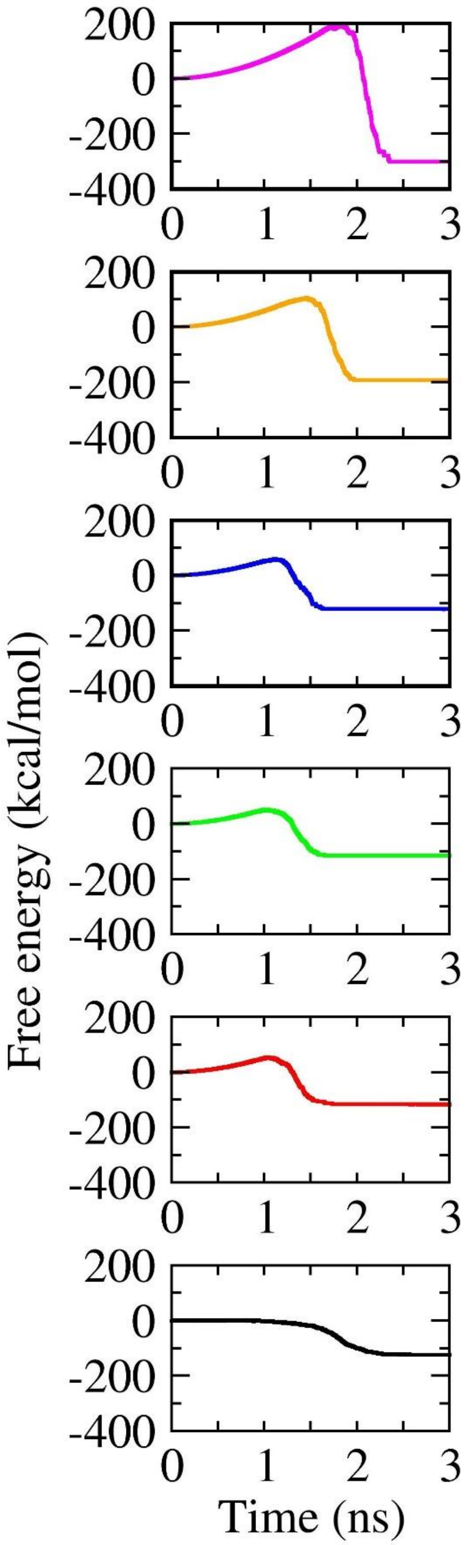

Figure S13: Free energy obtained from 100 independent trajectories of 4JNJ system, under 6 different restrained modes: mode 1 (in magenta); mode 2 (in orange); mode 3 (in blue); mode 4 (in green); mode 5 (in red) and mode 6 (in black).

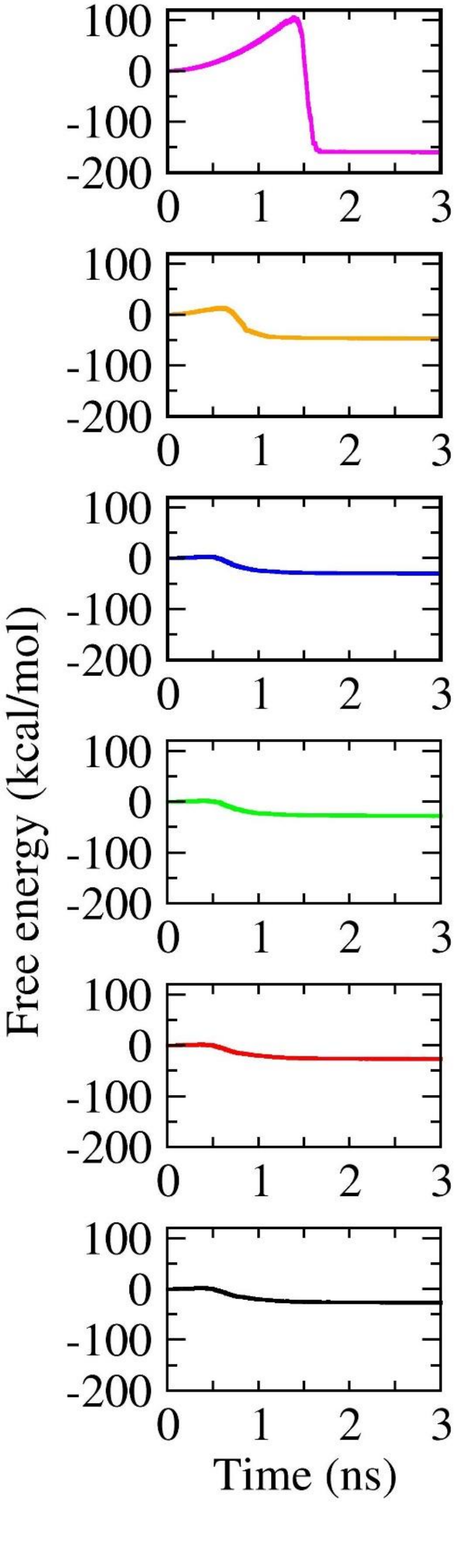

Figure S14: Free energy obtained from 100 independent trajectories of 2JFZ system, under 6 different restrained modes: mode 1 (in magenta); mode 2 (in orange); mode 3 (in blue); mode 4 (in green); mode 5 (in red) and mode 6 (in black).

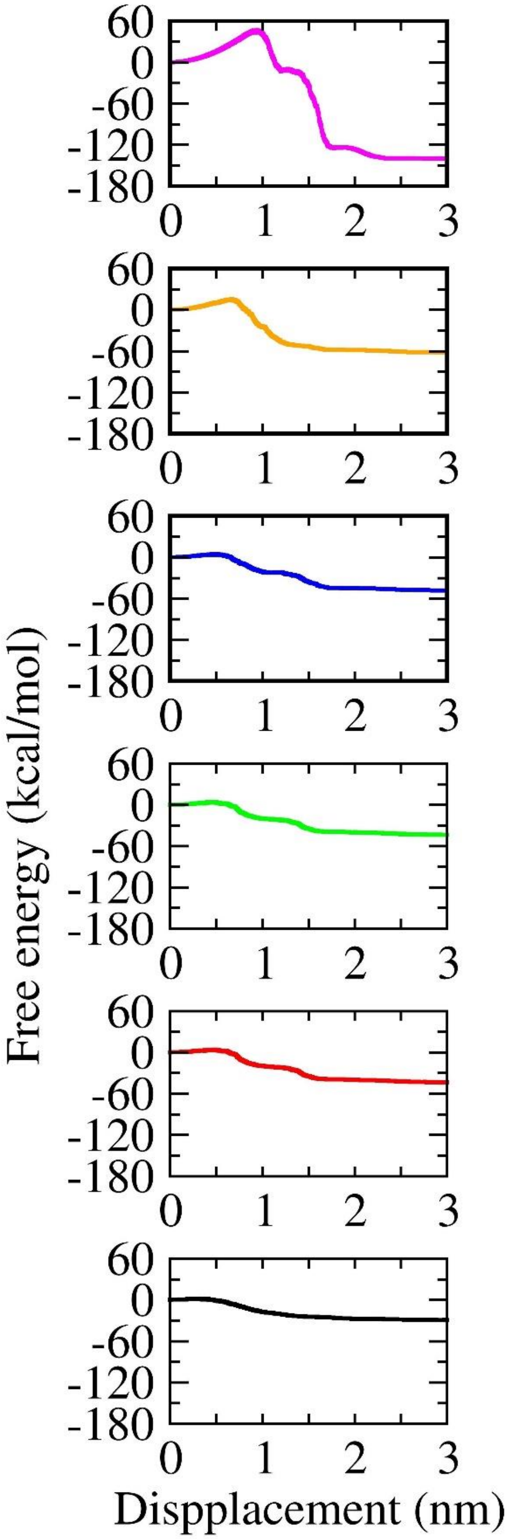

Figure S15: Free energy obtained from 100 independent trajectories of 1PYE system, under 6 different restrained modes: mode 1 (in magenta); mode 2 (in orange); mode 3 (in blue); mode 4 (in green); mode 5 (in red) and mode 6 (in black).

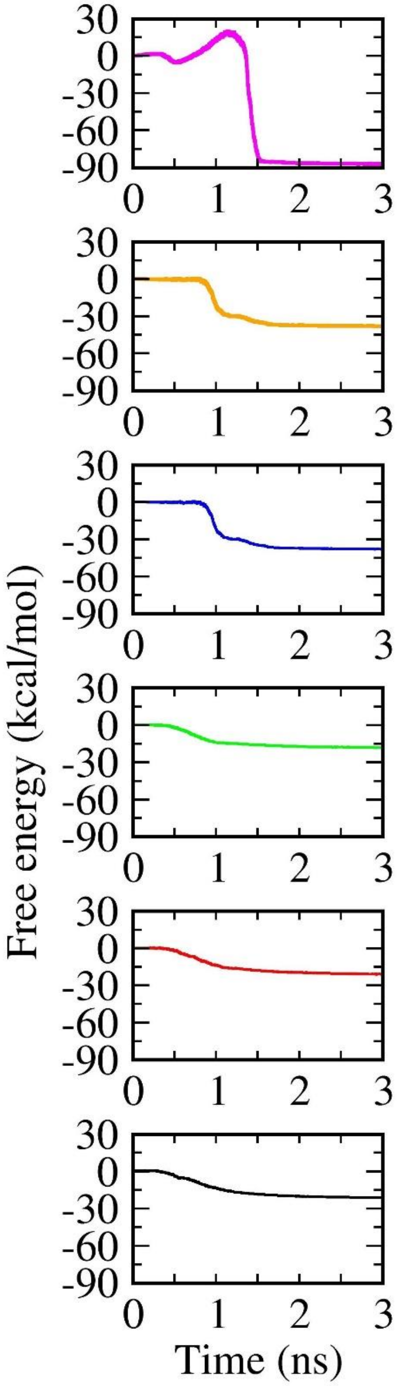

Figure S16: Free energy obtained from 100 independent trajectories of 1TSL system, under 6 different restrained modes: mode 1 (in magenta); mode 2 (in orange); mode 3 (in blue); mode 4 (in green); mode 5 (in red) and mode 6 (in black).

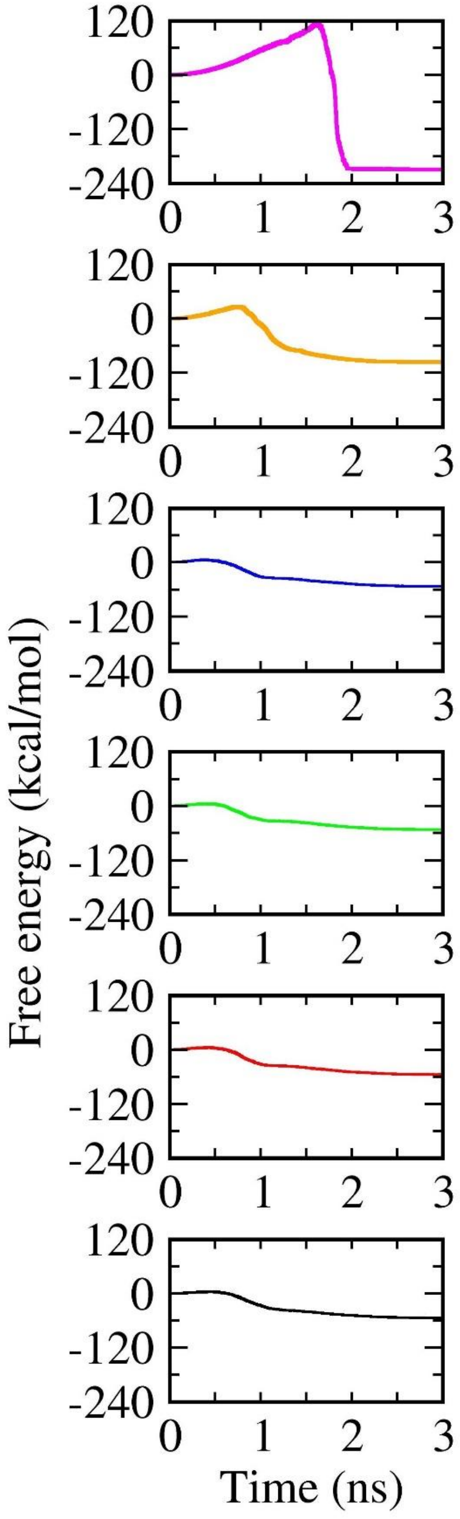

Figure S17: Free energy obtained from 100 independent trajectories of 2YDV system, under 6 different restrained modes: mode 1 (in magenta); mode 2 (in orange); mode 3 (in blue); mode 4 (in green); mode 5 (in red) and mode 6 (in black).

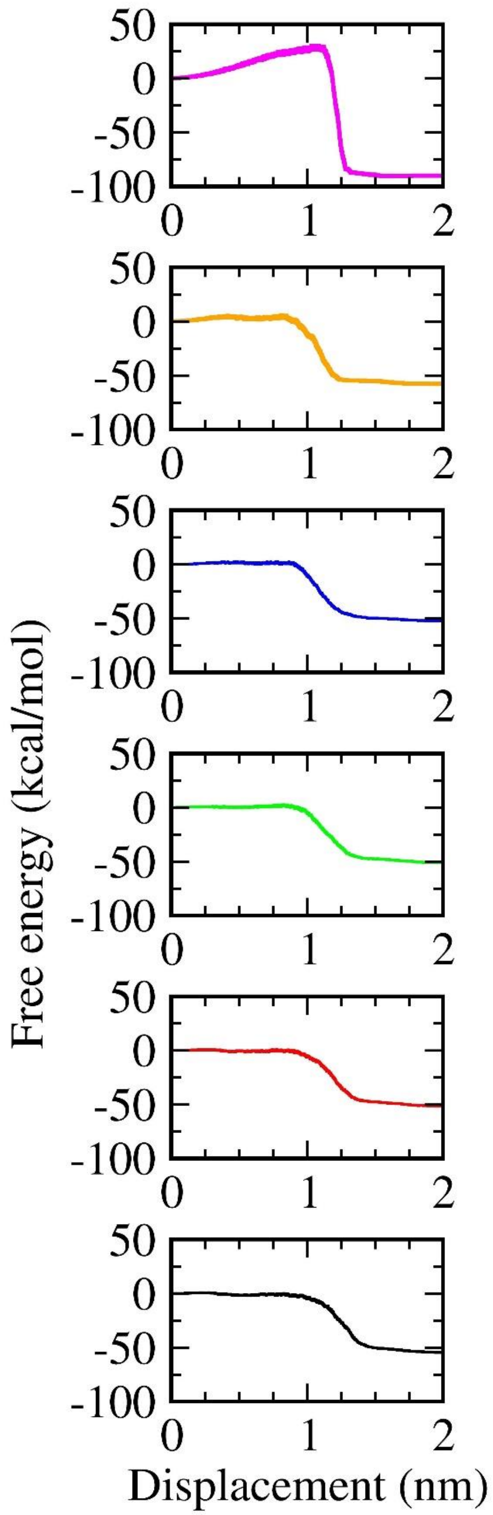

Figure S18: Free energy obtained from 100 independent trajectories of 1EVE system, under 6 different restrained modes: mode 1 (in magenta); mode 2 (in orange); mode 3 (in blue); mode 4 (in green); mode 5 (in red) and mode 6 (in black).

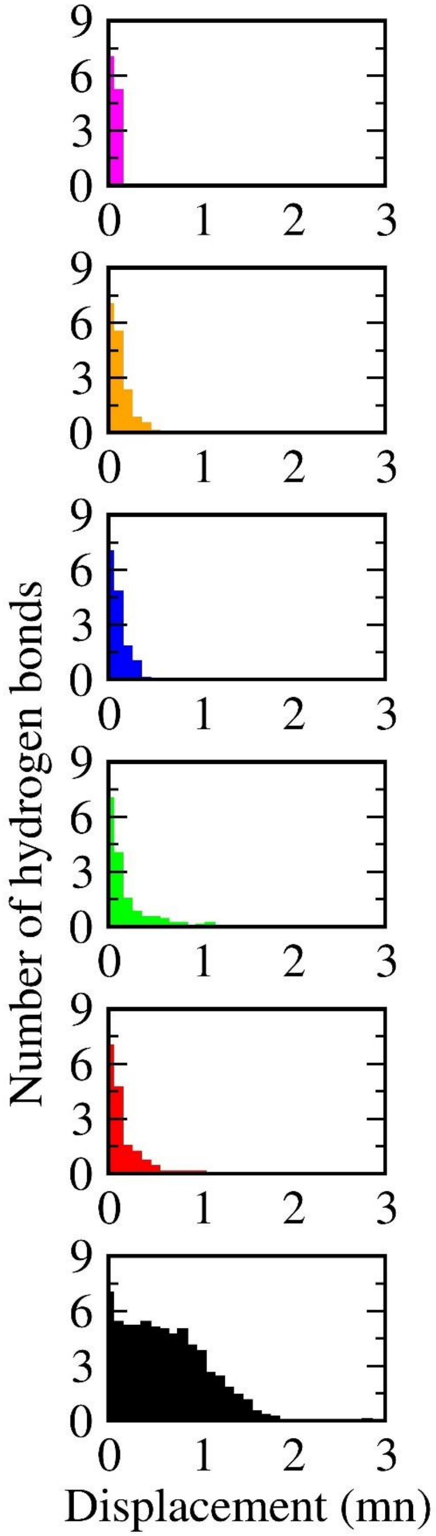

Figure S19: The averaging number of hydrogen bond in the dependence of displacement. Data was obtained from 100 independent trajectories of 4JNJ system, under 6 different restrained modes: mode 1

(in magenta); mode 2 (in orange); mode 3 (in blue); mode 4 (in green); mode 5 (in red) and mode 6 (in black).

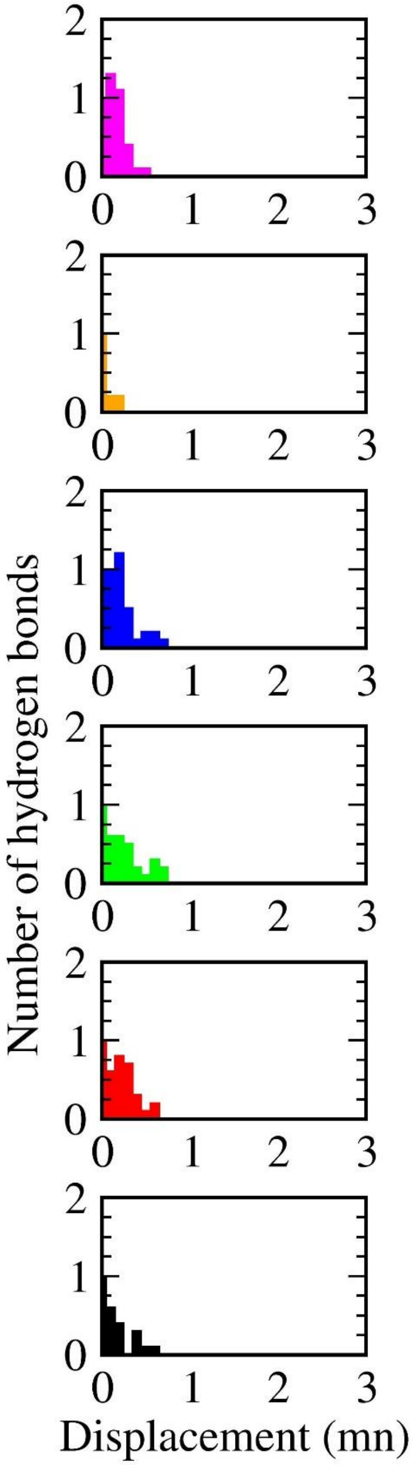

Figure S20: The averaging number of hydrogen bond in the dependence of displacement. Data was obtained from 100 independent trajectories of 2JFZ system, under 6 different restrained modes: mode 1

(in magenta); mode 2 (in orange); mode 3 (in blue); mode 4 (in green); mode 5 (in red) and mode 6 (in black).

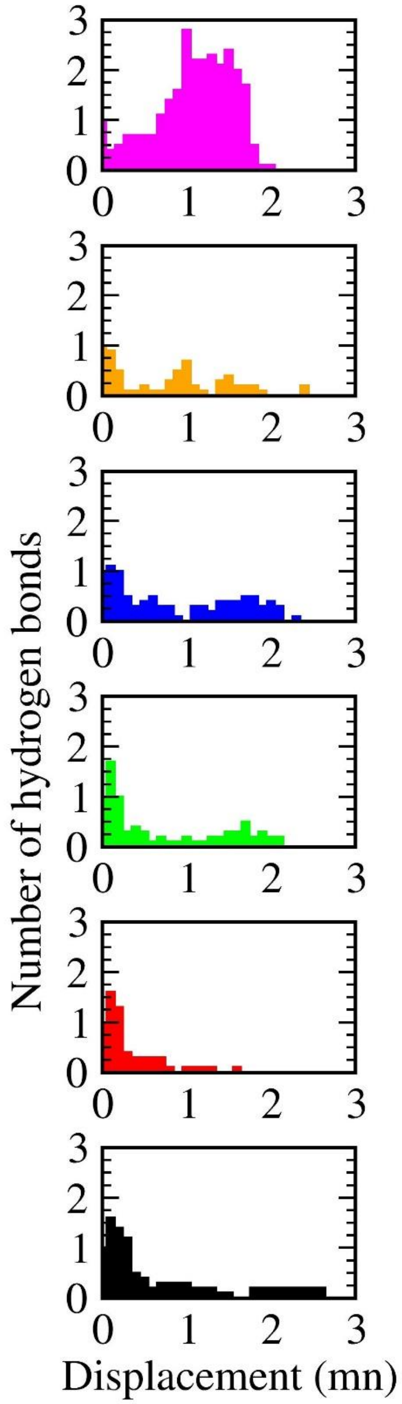

Figure S21: The averaging number of hydrogen bond in the dependence of displacement. Data was obtained from 100 independent trajectories of 1PYE system, under 6 different restrained modes: mode 1

(in magenta); mode 2 (in orange); mode 3 (in blue); mode 4 (in green); mode 5 (in red) and mode 6 (in black).

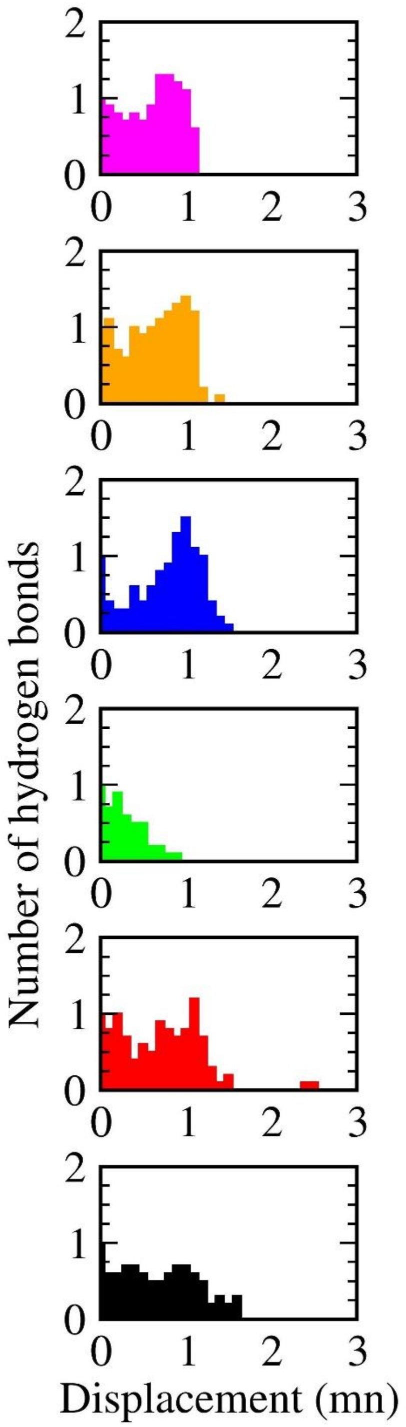

Figure S22: The averaging number of hydrogen bond in the dependence of displacement. Data was obtained from 100 independent trajectories of 1TSL system, under 6 different restrained modes: mode 1

(in magenta); mode 2 (in orange); mode 3 (in blue); mode 4 (in green); mode 5 (in red) and mode 6 (in black).

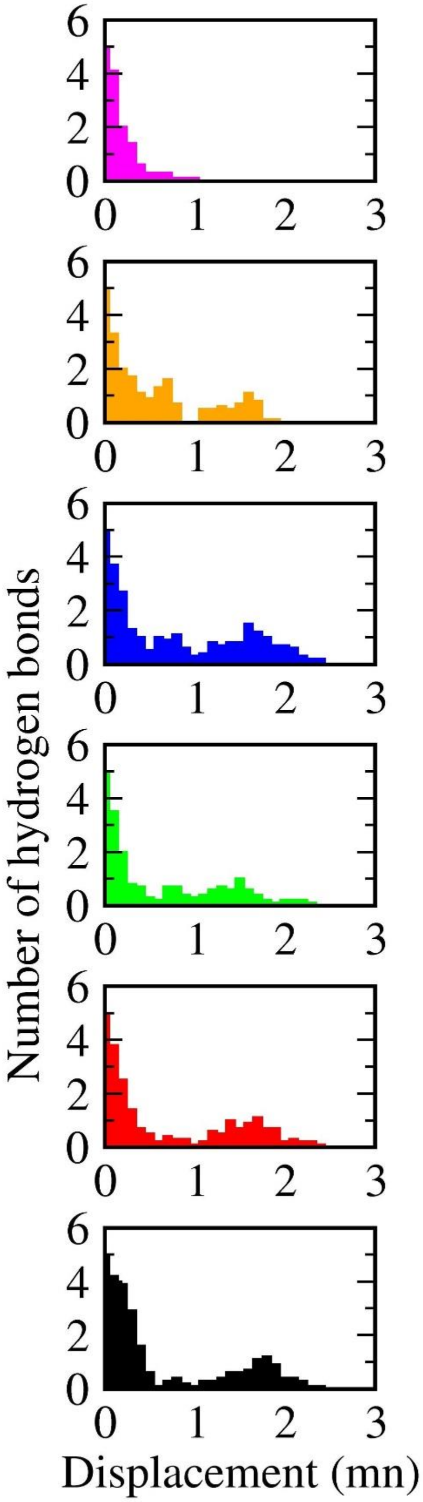

Figure S23: The averaging number of hydrogen bond in the dependence of displacement. Data was obtained from 100 independent trajectories of 2YDV system, under 6 different restrained modes: mode 1 (in magenta); mode 2 (in orange); mode 3 (in blue); mode 4 (in green); mode 5 (in red) and mode 6 (in black).

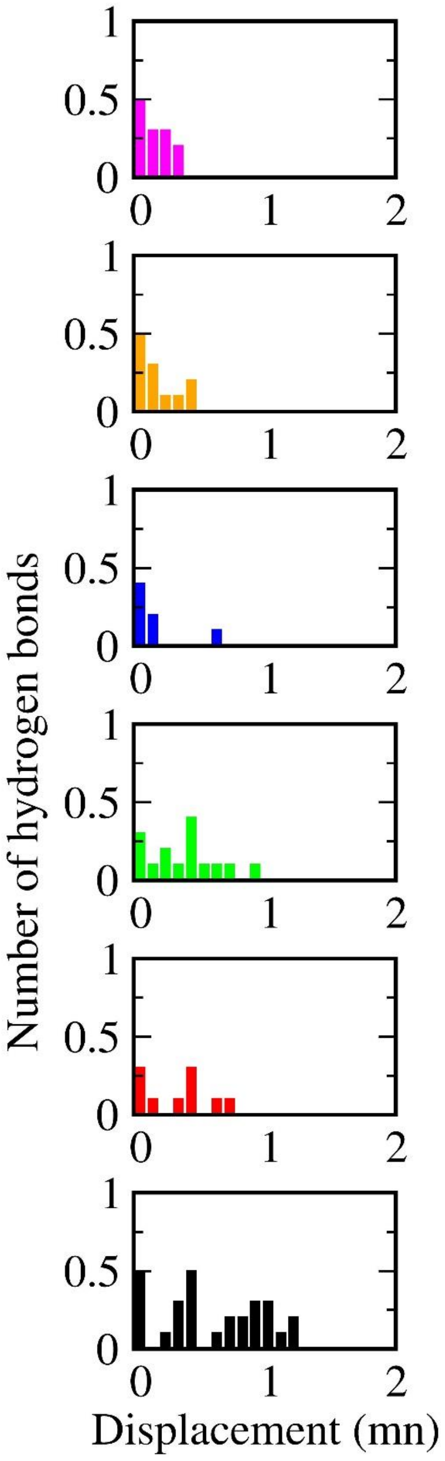

Figure S24: The averaging number of hydrogen bond in the dependence of displacement. Data was obtained from 100 independent trajectories of 1EVE system, under 6 different restrained modes: mode 1 (in magenta); mode 2 (in orange); mode 3 (in blue); mode 4 (in green); mode 5 (in red) and mode 6 (in black).

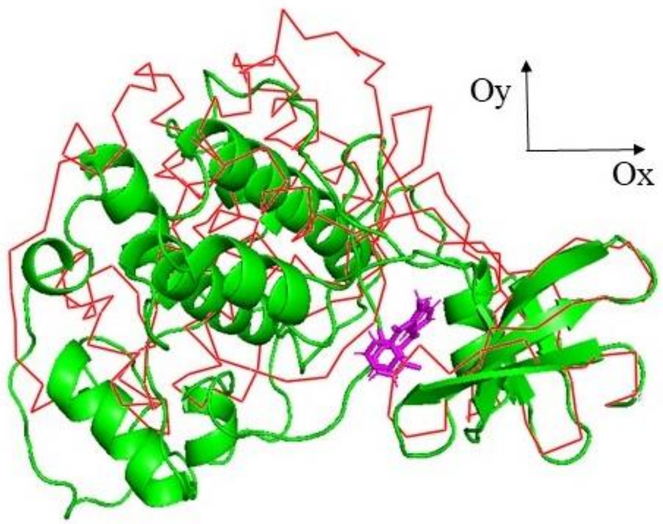

Figure S25*: In case of restraining few atoms, mode – 6 of 1PYE system, the protein is drifted. We plot two structure: a protein's initial structure (in green cartoon) and a snapshot (in red line). The rotation of protein has induced a crash when ligand was moving in a chosen z – direction*

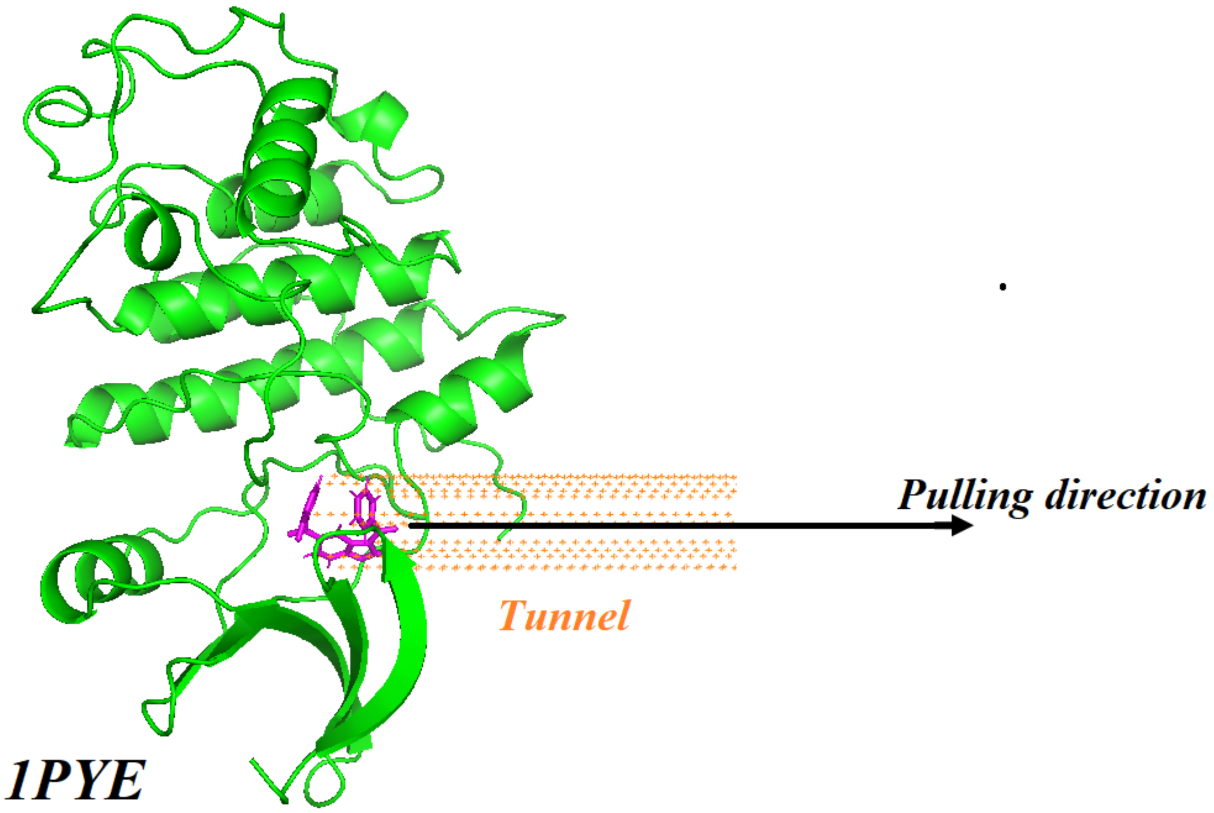
Pulling direction
Tunnel
1PYE

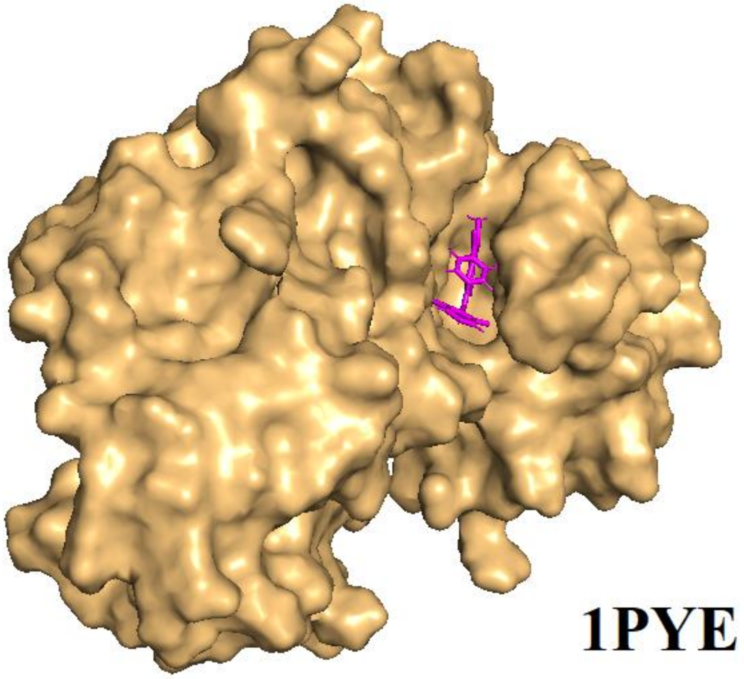
1PYE

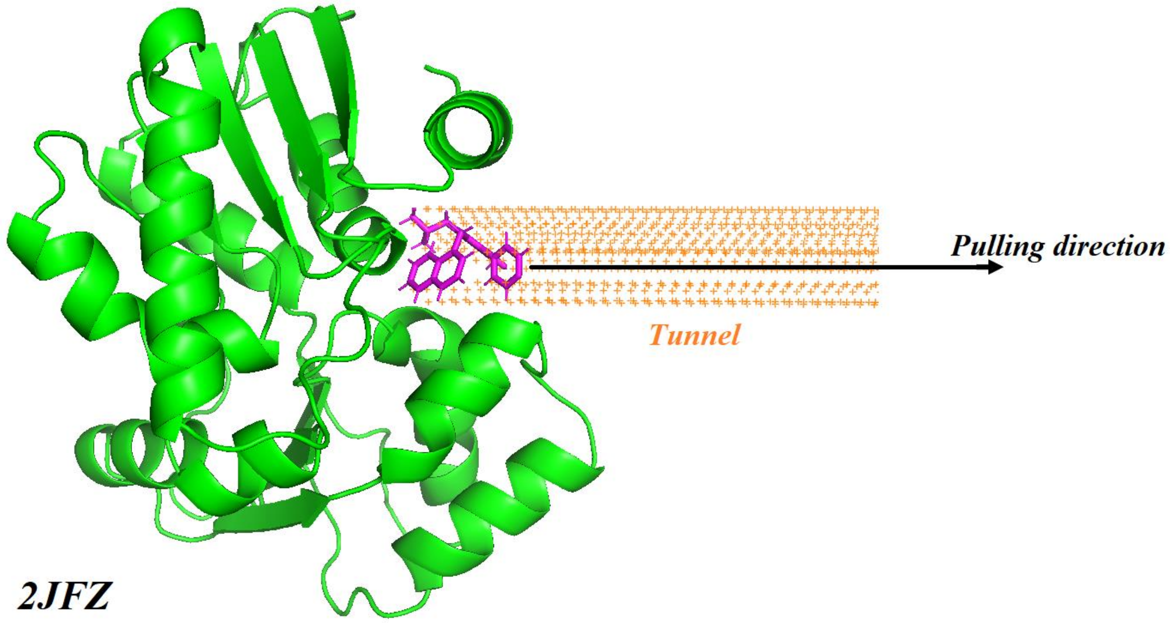
Pulling direction
Tunnel
2JFZ

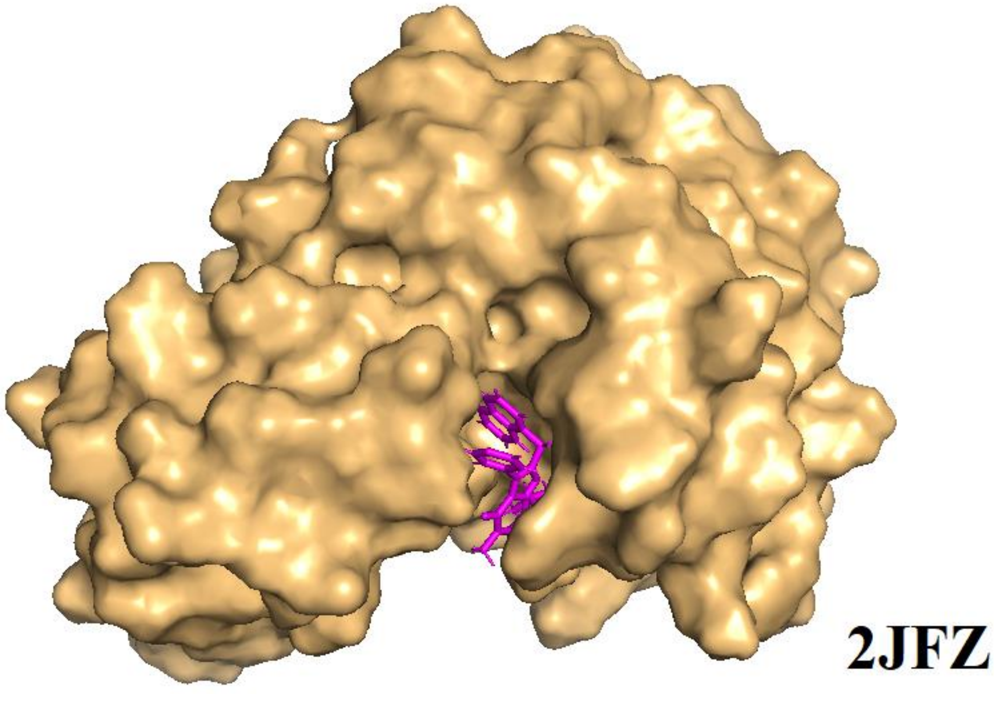
2JFZ

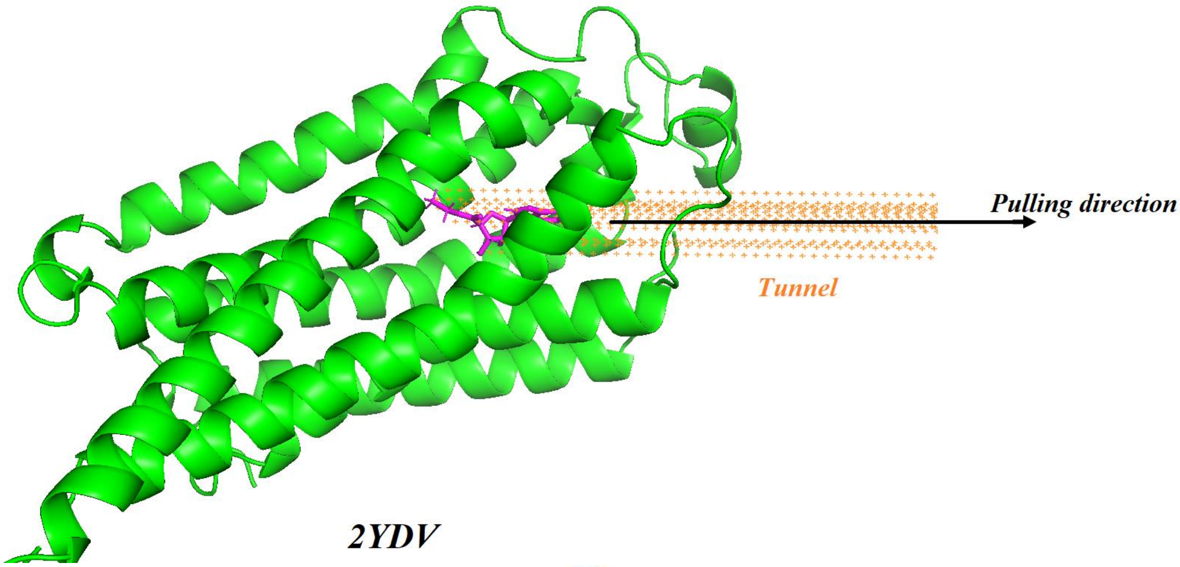
Pulling direction
Tunnel
2YDV

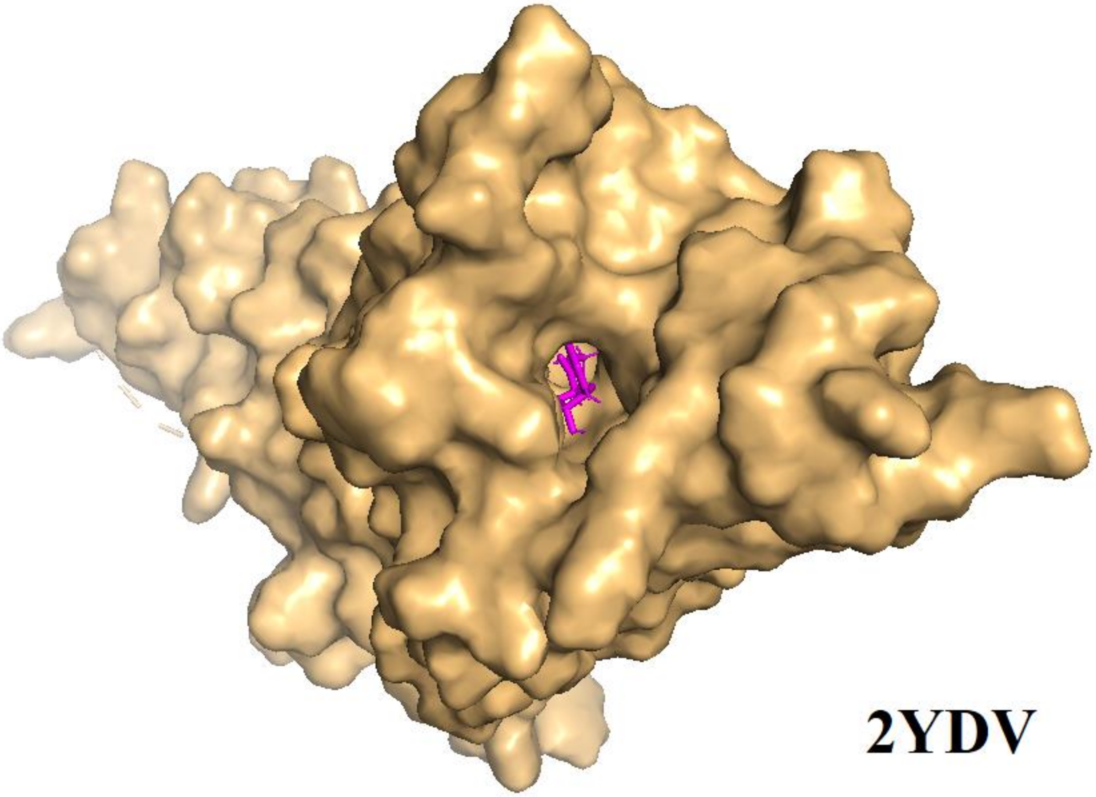
2YDV

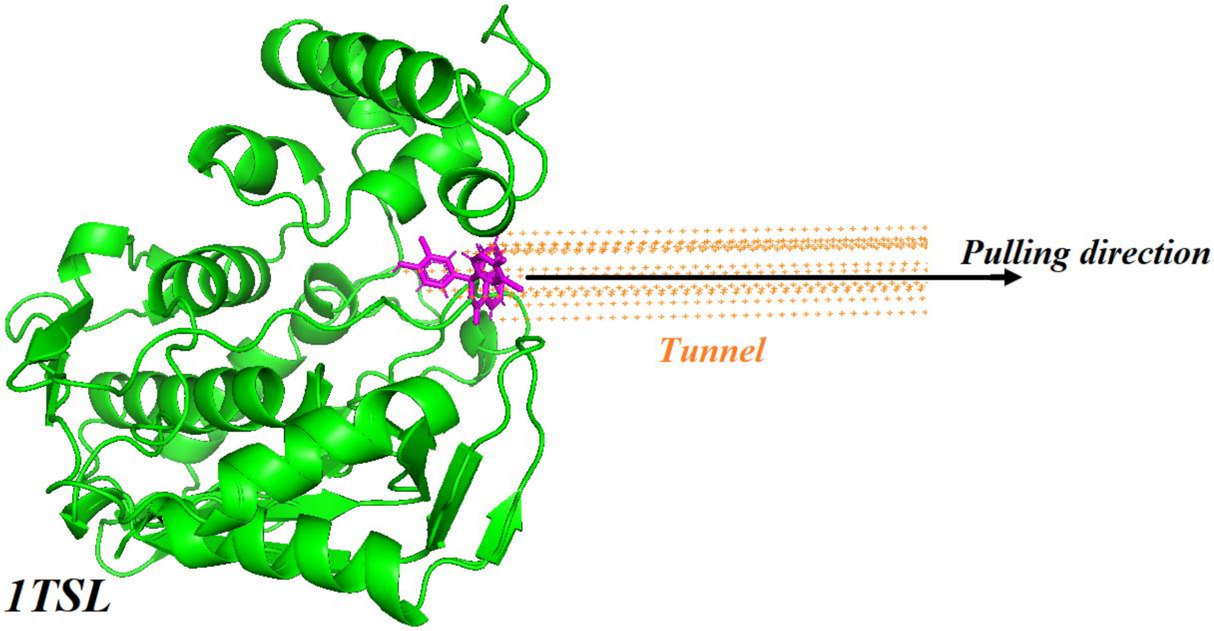

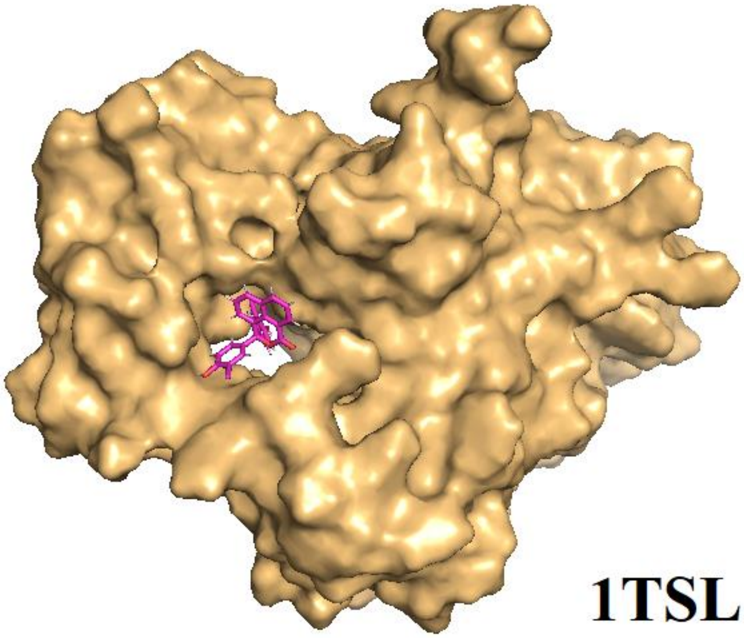

1TSL

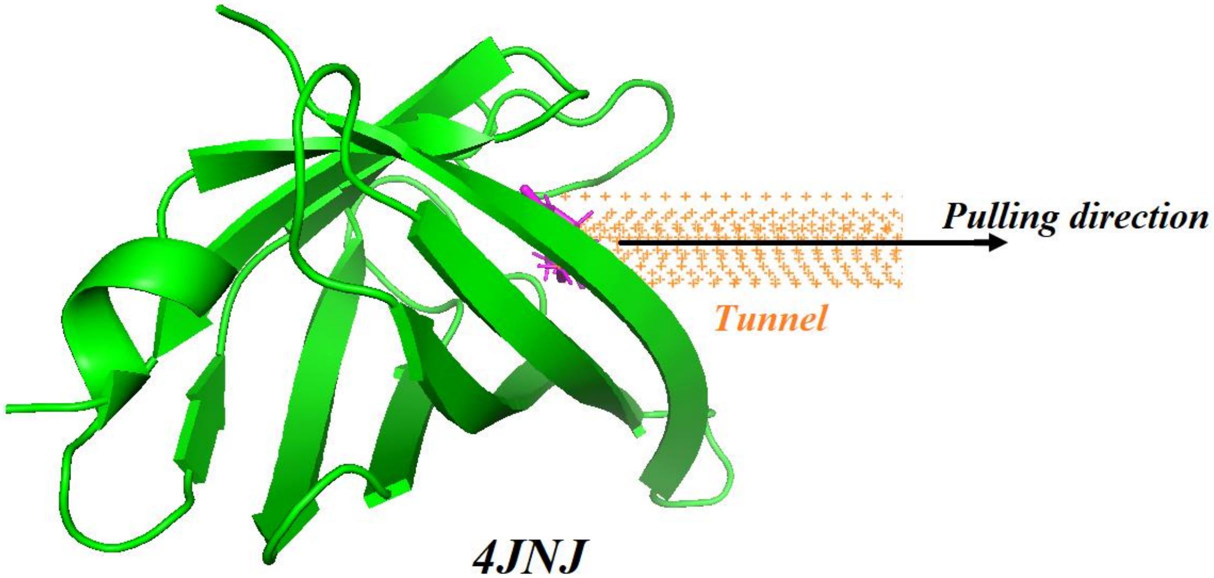

4JNJ

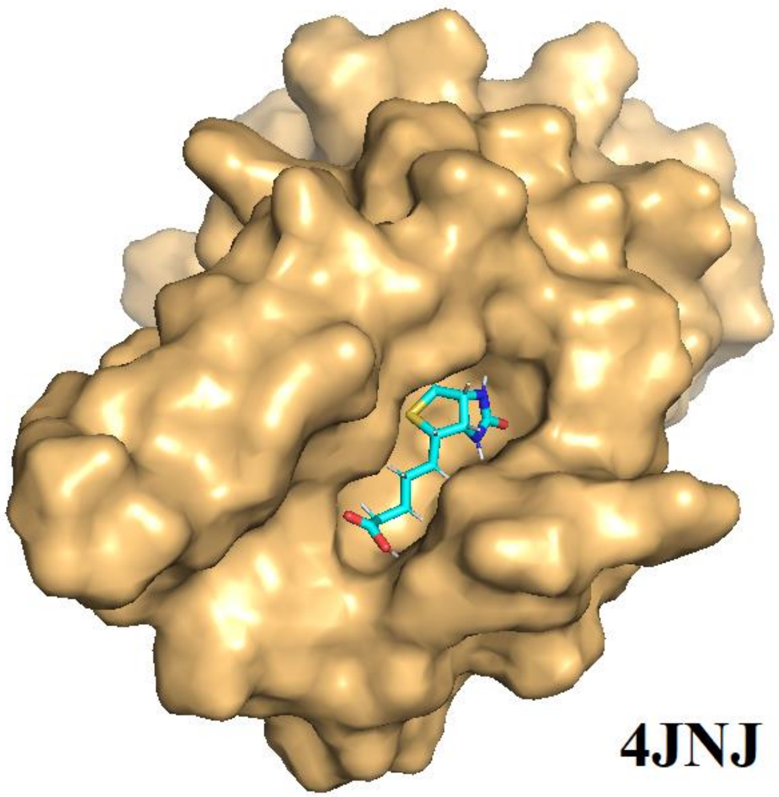

4JNJ

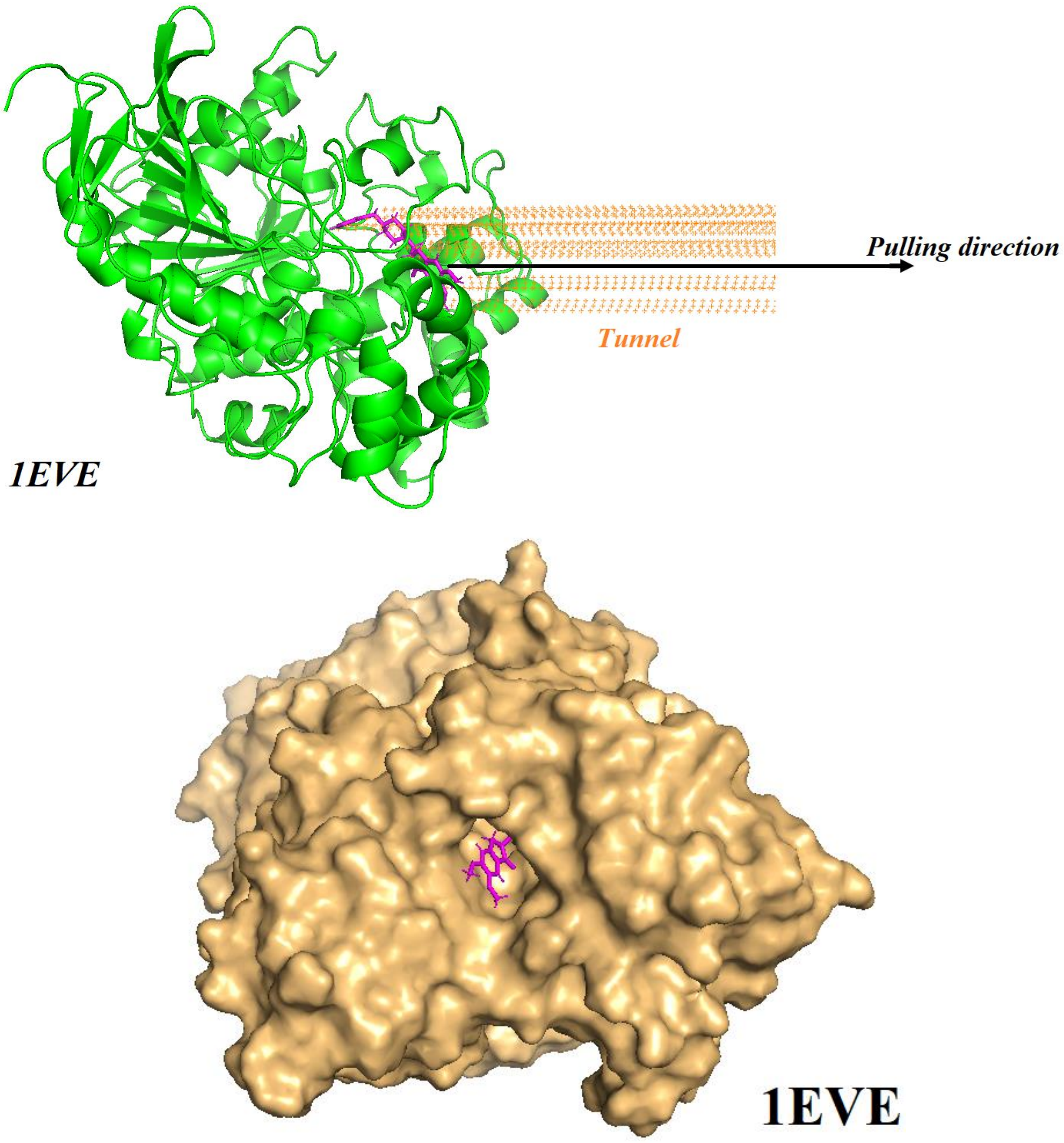

Figure S26: the pockets surface, escape tunnel and pulling direction of 6 complexes in front view and side view.
